# Supplementary material for: Activity-based protein profiling of human and plasmodium serine hydrolases and interrogation of potential antimalarial targets
Source: iScience. 2022 Aug 24;25(9):104996. doi: 10.1016/j.isci.2022.104996 (PMC9464883; doi:10.1016/j.isci.2022.104996)
Supplement: Document S1. Figures S1, S2, and Table S2 [file mmc1.pdf]

iScience, Volume *25*

Supplemental information

Activity-based protein profiling of human and *plasmodium* serine hydrolases and interrogation of potential antimalarial targets

Dara Davison, Steven Howell, Ambrosius P. Snijders, and Edgar Deu

Supplementary Material

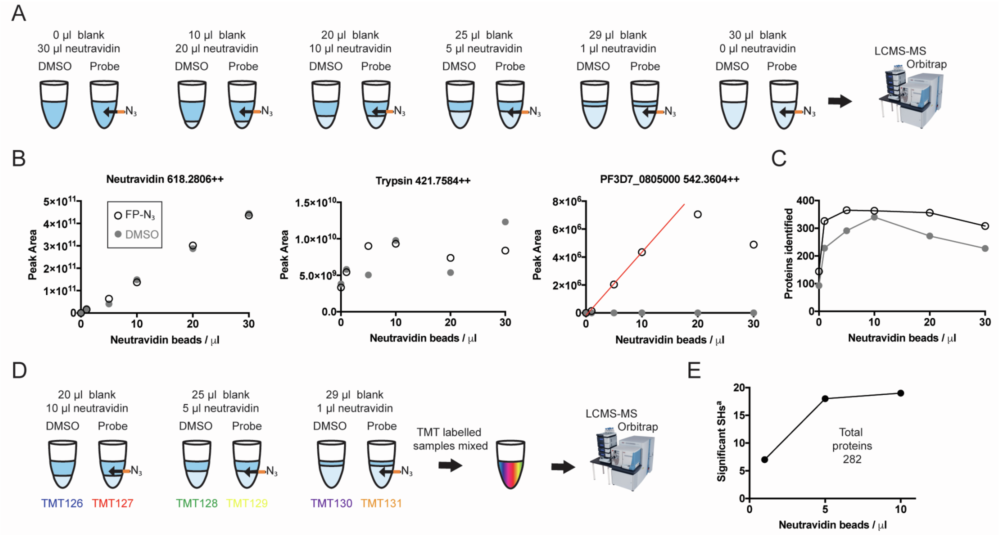

Figure S1. Optimisation of affinity purification for chemical proteomics, related to Figure 2. Contamination from tryptic neutravidin and trypsin peptides is inherent the proteomics methods and can mask identification of lower abundance peptides. We proposed to reduce the ratio of neutravidin to non-neutravidin peptides by optimising the volume of NBs used in order to achieve the highest bead engagement with the lowest bead volume. **(A)** Schizont lysates were labelled in triplicate with 1 μM FP-N_3 or DMSO and treated with CuAAC-chemistry reagents. Labelled proteins were affinity purified using six different NB:blank beads mixtures, followed by on-bread trypsin digestion and LC-MS/MS analysis. **(B)** The raw intensities of specific peptides from neutravidin, trypsin and SHs were assessed using Skyline. In probe-treated samples, neutravidin peptides increased in intensity linearly with NB volume (shown by red line), but the increase in SH peptide intensity slowed above 10 μl NB. Trypsin peptide intensities were not as constant as expected but showed no clear trend. **(C)** Proteins were then quantified using iBAQ and filtered as previously described. In probe-treated samples, the number of total proteins and SHs identified plateaued at 5 μl. Because most SHs were not detected in the DMSO control, the fold enrichment could only be calculated for a small fraction of the SHs identified in the probe-treated samples. **(D)** In order to circumvent the problem of low SH identifications in the control samples and improve our quantification method, we tested TMT isobaric labels to re-quantify the 1, 5 and 10 μl NB samples (TMT-sixplex) as these conditions gave high total protein IDs with low raw intensities of neutravidin peptides (**B-C**). TMT labelled samples are subsequently combined, and the mixture is fractionated by high pH before LCMS-MS. **(E)** Fewer total proteins were identified in the TMT samples, but the number of proteins identified (282) was consistent over all FP-N_3- and DMSO-treated samples, as expected. The highest number of SHs significantly enriched in FP-N_3- vs DMSO-treated samples ([a]student t-test, FDR = 0.05, s_0 = 0.5) was found with 10 μl NB.

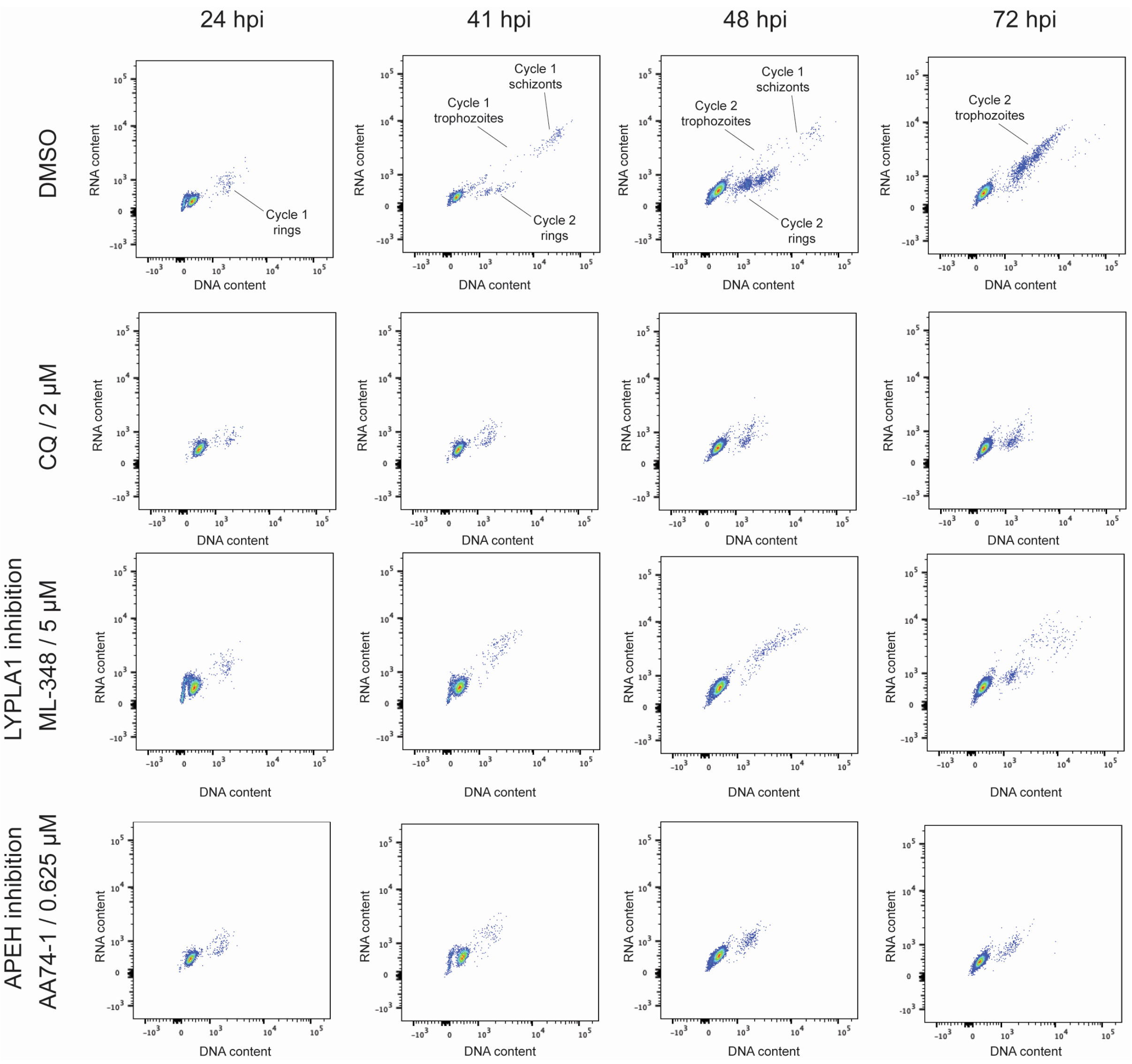

Figure S2. Replication assay to monitor the effect of human SH inhibitors on parasite growth and replication, Related to Figure 5. A growth assay was carried out in the presence of inhibitors ML348 or AA74-1 (3 times the calculated EC_{50}), DMSO and CQ were used as negative and positive controls. Parasites were treated at ring stage (0 hpi). At 24, 41, 48 and 72 hpi, samples were fixed and stained with Hoechst and the RNA dye 132A. This experiment was performed in triplicate. A representative FACS plot for each treatment and time point is shown. Flow cytometry plots show changes in DNA and RNA level overtime for each treatment. The population of RBCs with low DNA and RNA signal correspond to uRBCs. The three different morphological stages of the parasites in iRBCs can be clearly identified as: rings, low DNA and RNA content; trophozoites, low DNA, medium RNA content, and schizonts, high DNA and RNA content. DMSO-treated parasites developed as expected. CQ- and AA74-1-treated samples were arrested at ring stage. ML348-treated parasites appear to still be in trophozoite stage at 48 hpi. While DMSO treated parasite are actively egressing and invading at 48 hpi, no rings were detected in ML438-treated samples until 72 hpi.

Table S1. Activity-based protein profiling across the *Plasmodium falciparum* asexual lifecycle data, Related to Figure 3.
Excel file

Table S2. Primers for the generation of *P. falciparum* conditional mutant lines, related to Key resource table, Oligonucleotides.

Primer	Sequence (5’-3’)	Source	Identifier
Colony and 3’ integration PCR F 119	ACAGCTATGACCATGATTACGC	Sigma	N/A
Colony PCR and synthetic DNA amplification R 256	CGAATAGCCTCTCCACCCAA	Sigma	N/A
Excision PCR R 198	CCTCTCCACTGACAGAAAATTTGTGCC	Sigma	N/A
Synthetic DNA amplification F 257 (also abH114 excision PCR F)	TGGAGAGGCTATTCGGCTATGAC	Sigma	N/A
abH112 3' integration PCR R 296	AGCATTTTCAACTCCCCAGAATCGAAG	Sigma	N/A
abH112 Amplification and mutation R 270	CTTCCTCTTCCTTCTCCGTCGACCTCGTTC	Sigma	N/A
abH112 Endogenous PCR F 315	CATTCTTGGTTGTATGACCATATGGTAG	Sigma	N/A
abH112 Endogenous PCR R 317	ATTCTATATAATTCATGTAACAGTAACATC	Sigma	N/A
abH112 Excision PCR F 295	GAATACTACAGATATTGGTTATATGACAC	Sigma	N/A
abH112 HR amplification F 284	AGACTTAGGAGGAGATCTGTAGCGGGTTTAGGGGACGCATGGTGAA	Sigma	N/A
abH112 HR amplification R 269	CTTAATCCATAACCACTATAATCATATGAA	Sigma	N/A
abH112 Mutation PCR amplification 2/2 SER a R 278	CCAAGAGAATGTCCATAAACAATAATATTC	Sigma	N/A
abH112 Mutation PCR amplification 2/2 SER b F 277	TGGACATTCTCTTGGATCTGCAACCAGTTGTTA	Sigma	N/A
abH112 Synthetic DNA amplification ½ F 271	GTGGTTATGGATTAAGTAATAAAGATCCTAGTG	Sigma	N/A
abH114 3' integration PCR R 299	GGAATTCTCTAAAATGATTTGGCTAGCTGAAGG	Sigma	N/A
abH114 Amplification and mutation R 276	AATAAACTTCCTCTTCCTTCTCCGTCGACTTTG	Sigma	N/A
abH114 Endogenous PCR F 319	CAGTTATATACCTTTATCAGGTGTGT	Sigma	N/A
abH114 Endogenous PCR R 321	CATACATATAAATCTTCTTTATTATGC	Sigma	N/A
abH114 HR amplification F 283	AGACTTAGGAGGAGATCTTCCCCTTATAAATCTTTCAGAAAGAACGTC	Sigma	N/A
abH114 HR amplification R 274	ATCGCATATTTTCGGTATGCTTAGAATC	Sigma	N/A
abH114 Mutation PCR amplification 2/2 SER a R 281	CCTCCTAAAGAGAATCCAATTAAGCAAC	Sigma	N/A
abH114 Mutation PCR amplification 2/2 SER b F 280	GATTCTCTTTAGGAGGAAAAGCCTCTAT	Sigma	N/A
abH114 Synthetic DNA amplification ½ F 275	CCGAAAATATGCGATTTGATGAAATTG	Sigma	N/A
Psta1 5' integration PCR R 291	GTCCATATTCTCCAATGTATATATCTCC	Sigma	N/A
Psta1 Amplification and mutation R 235	TTCCTTCTCCGTCGACTTCA	Sigma	N/A
Psta1 Endogenous PCR R 309	CATTTAATTATATTCTAAAGACTTTG	Sigma	N/A
Psta1 Endogenous/5’ excision PCR F 307	CTTACTATAATAATAAAGTGAACACACA	Sigma	N/A
Psta1 Excision PCR F 289	AGTACATGGGTTAGCTTCTCATATAAGAT	Sigma	N/A
Psta1 HR amplification F 218	ACTTAGGAGGAGATCTAATAATAAAAATGATATAACCATTGATGAGAA	Sigma	N/A
Psta1 HR amplification R 225	CTATAATATCGTTATCTCTTATATACATTTGTTT	Sigma	N/A
Psta1 Mutation PCR amplification 2/2 SER a R 240	ATACTATAACCAACAAGGTATATTGGTA	Sigma	N/A
Psta1 Mutation PCR amplification 2/2 SER b F 239	TTGGTTATAGTATGGGAGCTAATATAATACTTAGGGC	Sigma	N/A
Psta1 Synthetic DNA amplification ½ F 223	ATAACGATATTATAGGTAAATAAAAAAAAATAATATACAATAACTTCGTATAGCATACAT	Sigma	N/A
Psta2 5' integration PCR R 294	CTTAGTTATTGTACCGTCGTACCTCACTTT	Sigma	N/A
Psta2 Amplification and mutation R 236	TTCCTTCTCCGTCGACTTTT	Sigma	N/A
Psta2 Endogenous PCR R 313	AAACGATTCAGTGTAGACAAA	Sigma	N/A
Psta2 Endogenous/5’ excision PCR F 311	TTACACTGTTGTATAAGTTTGAA	Sigma	N/A
Psta2 Excision PCR F 292	TACATGGGTTAGCTTCTCATTTAAGG	Sigma	N/A
Psta2 HR amplification F 220	ACTTAGGAGGAGATCTACAGAAAGTAATATTATTAATGATGAGAA	Sigma	N/A
Psta2 HR amplification R 229	CTGCTCCTTATCTAATGTGTCACTTCTCGTTTC	Sigma	N/A
Psta2 Mutation PCR amplification 2/2 SER a R 242	ACCCATATCAAAACCAGCCAAGTAAATTG	Sigma	N/A
Psta2 Mutation PCR amplification 2/2 SER b F 241	GGTTTTAGTATGGGTGCCAATATTATGTTACG	Sigma	N/A
Psta2 Synthetic DNA amplification ½ F 232	TTAGATAAGGAGCAGGTAAATAAAAAAAAATAATATACAATAACTTCGTATAGCATACAT	Sigma	N/A
