# Supplementary material for: Activity-based protein profiling of human and plasmodium serine hydrolases and interrogation of potential antimalarial targets
Source: iScience. 2022 Aug 24;25(9):104996. doi: 10.1016/j.isci.2022.104996 (PMC9464883; doi:10.1016/j.isci.2022.104996)
Supplement: Document S2. Article plus supplemental information [file mmc3.pdf]

iScience

CellPress
OPEN ACCESS

Article

Activity-based protein profiling of human and *plasmodium* serine hydrolases and interrogation of potential antimalarial targets

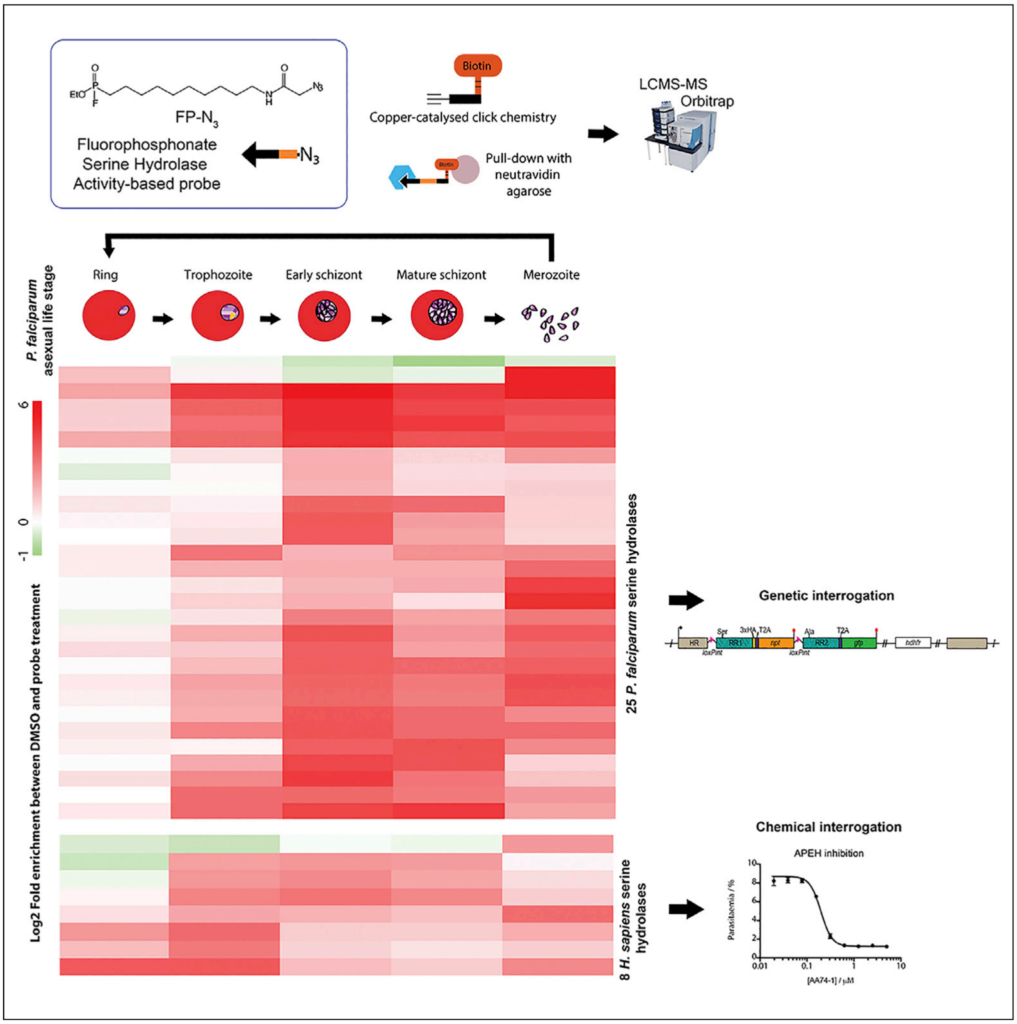

Dara Davison, Steven Howell, Ambrosius P. Snijders, Edgar Deu

dara.davison@crick.ac.uk

Highlights

P. falciparum has 48 predicted metabolic SHs. Many react with the ABP, FP-N_3

The activity of 25 *Pf*SHs and 8 *Hs*SHs was profiled throughout the asexual life cycle

Catalytic mutants of 4 *Pf*SHs (formerly held essential) had no parasite growth effect

Selective inhibitors for 2 *Hs*SHs (APEH and LPLA2) affected parasite growth

Davison et al., iScience *25*, 104996
September 16, 2022 © 2022 The Authors.
https://doi.org/10.1016/j.isci.2022.104996

Check for updates

iScience

CellPress OPEN ACCESS

Article

Activity-based protein profiling of human and *plasmodium* serine hydrolases and interrogation of potential antimalarial targets

Dara Davison,[1,3,*] Steven Howell,[2] Ambrosius P. Snijders,[2] and Edgar Deu[1]

SUMMARY

Malaria remains a global health issue requiring the identification of novel therapeutic targets to combat drug resistance. Metabolic serine hydrolases are druggable enzymes playing essential roles in lipid metabolism. However, very few have been investigated in malaria-causing parasites. Here, we used fluorophosphonate broad-spectrum activity-based probes and quantitative chemical proteomics to annotate and profile the activity of more than half of predicted serine hydrolases in *P. falciparum* across the erythrocytic cycle. Using conditional genetics, we demonstrate that the activities of four serine hydrolases, previously annotated as essential (or important) in genetic screens, are actually dispensable for parasite replication. Of importance, we also identified eight human serine hydrolases that are specifically activated at different developmental stages. Chemical inhibition of two of them blocks parasite replication. This strongly suggests that parasites co-opt the activity of host enzymes and that this opens a new drug development strategy against which the parasites are less likely to develop resistance.

INTRODUCTION

Despite a significant drop in incidence over the last two decades, malaria remains a global health problem with 229 million estimated cases and close to half a million deaths in 2019 (WHO, 2020). Malaria is caused by parasites of the *Plasmodium* genus and is transmitted by *Anopheles* mosquitoes. *Plasmodium falciparum* is the most virulent species responsible for 90% of mortality. Malaria control has been hampered by the rise of insecticide-resistant mosquitoes and drug-resistant parasites (WHO, 2018). It is therefore imperative to develop new therapies to treat malaria. To slow down the emergence of resistance, new drug candidates should have a novel mechanism of action (Burrows et al., 2013, 2017). *Plasmodium* parasites have a complex life cycle that involves sexual replication in the mosquito host and asexual replication in humans. In the human blood stream, parasites replicate exponentially inside red blood cells (RBCs). This asexual erythrocytic cycle is responsible for the vast majority of malaria pathology. Therefore, new antimalarial drugs need to be highly effective at this stage, but an ideal treatment should also kill other developmental stages to block and prevent transmission.

Serine hydrolases (SHs) are a large family of druggable targets that remain largely unexplored in *Plasmodium* species. The SH superfamily includes all enzymes that use a nucleophilic serine to catalyze the hydrolysis of bonds, such as amide, ester, or thioester groups. These reactions proceed by a conserved catalytic mechanism, i.e., formation of an acyl-enzyme intermediate that undergoes water-induced hydrolysis to release the products. We can take advantage of this common mechanism to design broad-spectrum irreversible inhibitors and probes (Bachovchin and Cravatt, 2012). SHs are involved in many biological processes such as metabolism, post-translational modification, and proteolysis. Metabolic SHs are ubiquitous in all organisms and important in almost all human diseases (Long and Cravatt, 2011); for example, acetylcholinesterase in neurotransmission (Lane et al., 2006), phospholipase A2 in inflammation (Bonventre et al., 1997), and lipases in bacterial infection (Shao et al., 2007). Serine proteases have been extensively studied in malaria and pursued as drug targets (Deu, 2017) but only a few metabolic SHs have been characterized: Pro-drug resistance esterase PARE (Istvan et al., 2017), Bud

[1]Chemical Biology Approaches to Malaria Laboratory, The Francis Crick Institute, London NW1 1AT, UK

[2]Mass Spectrometry Proteomics Science Technology Platform, The Francis Crick Institute, London NW1 1AT, UK

[3]Lead contact

*Correspondence: dara.davison@crick.ac.uk

https://doi.org/10.1016/j.isci.2022.104996

This is an open access article under the CC BY license (http://creativecommons.org/licenses/by/4.0/).

Emergence 46 protein (BEM46, Kumar et al., 2013; Mercker et al., 2009) and PNPLA1 (Flammersfeld et al., 2020; Singh et al., 2007). Sixty percent of mammalian metabolic SHs have an α/β-fold (α/βHs), usually accompanied by a Ser-His-Asp catalytic triad (Holmquist, 2000). Based on structural predictions the *P. falciparum* genome encodes 43 α/βHs (Table 1) with 4 patatin domain-containing lipases (PLPs, Wilson and Knoll, 2018) and one amidase (Mailu et al., 2015).

One common biological function of SHs is in lipid metabolism and membrane biogenesis. RBCs perform very little *de novo* lipid biosynthesis, therefore *P. falciparum* parasites must synthesize and scavenge their own phospholipids (Asahi et al., 2005; Déchamps et al., 2010a, 2010b; Marks et al., 1960). Membrane dynamics are extremely important as the parasite replicates and undergoes morphological changes during the erythrocytic cycle, which can be divided into four stages: ring, trophozoite, schizont, and merozoite (Figure 1A). Invasion of RBCs by merozoites results in invagination of the RBC membrane and formation of the parasitophorous vacuole (PV), within which the parasite develops and replicates (Paul et al., 2015, Riglar et al., 2011). In ring stage (0–20 h post invasion, hpi) the host cell is remodeled by the secretion of parasite proteins into the RBC cytosol and membrane (Zuccala and Baum, 2011). This puts in place nutrient acquisition pathways and strategies to evade the host immune system that require the formation of large membrane structures such as Maurer's Clefts and the tubovesicular network (Sherling and van Ooij, 2016). Trophozoite stage (20–36 hpi) is a very metabolically active period characterized by hemoglobin digestion in the food vacuole, a pathway that liberates space within the RBC and provides a source of amino acids for protein synthesis (Elliott et al., 2008). Large vesicles known as cytostomes transport hemoglobin from the RBC cytosol into the food vacuole. The schizont stage (36–48 hpi) consists of asynchronous nuclear division, membrane biogenesis, and finally, cytokinesis to form 20–32 daughter merozoites (Bannister and Mitchell, 1989). The final step in the life cycle is merozoite egress from the infected RBC (iRBC), a process that is tightly regulated by kinases such as cGMP-dependent protein kinase PKG and proteases such as subtilisin-like protease 1 (SUB1, Blackman, 2008; Collins et al., 2013b). Eighty percent of genes expressed in this cycle do so in a cyclic manner (Bozdech et al., 2003). Given the changing metabolic requirements and membrane remodeling/biogenesis throughout the cycle, *Pf*SHs likely perform essential functions at different stages and require temporal expression and/or activation. The antiplasmodial natural product salinipostin A targets multiple *Pf*SHs and arrests parasite growth in schizont stage (Yoo et al., 2020).

The aims of this study are to profile active SHs throughout the erythrocytic cycle of *P. falciparum* using quantitative chemical proteomics and to evaluate some of the identified SHs as potential antimalarial targets. Activity-based probes (ABPs) specifically and covalently react with the key catalytic nucleophilic residue of an enzyme via an electrophilic warhead. Fluorophore and affinity tags within the probe can be used to visualize or pull-down modified targets. Because the warhead only reacts with the catalytic residue in a functional active site, ABPs discriminate between active and inactive forms of an enzyme. In activity-based protein profiling (ABPP), broad-spectrum ABPs are used to profile the activity of a whole enzyme family by taking advantage of its conserved catalytic mechanism. ABPP can be used to look at changes in enzyme activation during a biological process such as cell fate commitment, life cycle progression, or in response to external stimuli (Cravatt et al., 2008; Liu et al., 1999; Niphakis and Cravatt, 2014; Willems et al., 2014).

Fluorophosphonate (FP) ABPs have been proven to be highly efficient to profile and identify SHs in complex proteomes (Jessani et al., 2005; Liu et al., 1999; Okerberg et al., 2005; Patricelli et al., 2001) and to assess the specificity of inhibitors against all members of an enzyme family (Bachovchin et al., 2010; Kidd et al., 2001). FP-desthiobiotin was recently used to annotate 20 *Pf*SHs in schizont-stage protein lysates (Elahi et al., 2019b). Here, we used the azide conjugated ABP FP-N_3 (Figure 1B). Unlike FP-desthiobiotin, FP-N_3 is cell permeable, allowing us to profile SH activity in live parasites. After cell lysis, FP-N_3 can be functionalized with biotin via copper(I)-catalyzed alkyne-azide cycloaddition (CuAAC) chemistry to pull-down and identify the modified targets by MS. Using this method, we identified 25 *Pf*SHs, including 8 that were not identified in the previous study, and measured changes in their activity throughout the erythrocytic cycle. We also identified 8 active human SHs (*Hs*SHs) in iRBCs, and used inhibitors to investigate the possibility that their activity is important in parasite growth and replication. Finally, we used conditional genetics to evaluate the essentiality of four *Pf*SHs, and small molecule inhibitors to interrogate the role of *Hs*SHs in parasite development.

Table 1. Predicted SHs in the *P. falciparum* genome

	PF3D7 Gene ID	Name	Sub-family	Putative function	PBANKA *Gene ID* / *Plasmo*GEM annotation[a]	*piggyBac* screen annotation[b]	Knockout Studies	Life cycle ABPP	Proteomics[c]	Transcrip-tomics[d]
α/βHs	0702200		Pst-a	lysophospholipase		dispensable				R
	0709700	PfPARE	Pst-a	Esterase	1220300 / dispensable	dispensable	dispensable in asexual stages[e]	ES, MS[f]	G, M	S, G
	1038900		Pst-a	Phospholipase		essential		ES	G	T
	1476700	Psta1	Pst-a	lysophospholipase		low fitness	dispensable in asexual stages	M	G	G
	1476800	Psta2	Pst-a	lysophospholipase		essential	dispensable in asexual stages	ES	G	G
	0937200		Pst-a	Enzyme		low fitness			G	G
	0936700		Pst-a	α/β-hydrolase		essential				R
	1252600		Pst-a	Esterase		essential		ES[f]		R
	1401500		Pst-a	Esterase		essential		ES, M		R
	0102400	S33H	Pst-a / S33	α/β-hydrolase		essential			T	S
	0629300	LCAT / PfPL		PC-sterol acyltransferase	1128100/ dispensable	low fitness	liver stage disruption in *P. berghei*[g]	MS[f]	R	Sp
	0731800	S33G	S33	α/β-hydrolase		low fitness		MS	G	G, R
	1001400	PfXL1 / S33F	S33	exported lipase 1		low fitness	dispensable in asexual stages[h]		R	S
	1001600	PfXL2		exported lipase 2		essential	dispensable in asexual stages[h]	M[f]	S	T
	1328500	MLPL		α/β-hydrolase		dispensable		M[f]	S	T, S, Sp
	1441600			α/β-hydrolase	1305500	dispensable			S, Sp	S, Sp, G
	1427100		Lipase_3	α/β-hydrolase	1017500 / dispensable	dispensable		T		R, Sp, G
	0709900		Duf_900	α/β-hydrolase	1220500	essential			A	R
	1359900		Duf_900	α/β-hydrolase	1136100	dispensable			S, M, Sp	
	1205900		Duf_726	membrane protein	0604600	essential			S, M, G	
	1116000	RON4	Duf_676	serine esterase	0932000 / essential	low fitness			S	S
	0823400			α/β-hydrolase	0707200	essential				G
	1142900	S33C	S33	α/β-hydrolase	0906100 / slow	essential			S, Sp	R, G, Sp
	0301300	EH1 / S33E	S33	α/β-hydrolase		low fitness	dispensable in asexual stages[i]	ES[f]		R, S
	0826200	S33B	S33	α/β-hydrolase	0704400 / dispensable	essential			Sp	S, G
	1401300	EH2 / S33D	S33	Aminopeptidase		low fitness	dispensable in asexual stages[i]	[f]	G	T, G
	1410100	S33A	S33	α/β-hydrolase	1032400 / dispensable	essential			G	G
	0728700			α/β-hydrolase	0212800	essential		[f]	T, Sp	G
	1120400	abH112		α/β-hydrolase		essential	dispensable in asexual stages	ES[f]		R

(*Continued on next page*)

Table 1. *Continued*

	PF3D7 Gene ID	Name	Sub-family	Putative function	PBANKA *Gene ID* / *Plasmo*GEM annotation[a]	*piggyBac* screen annotation[b]	Knockout Studies	Life cycle ABPP	Proteomics[c]	Transcrip-tomics[d]
	0403800	RhoSH	S9	α/β-hydrolase	1001500 / slow	essential	growth defect in asexual stages[j]	M[f]	S, M	S, G
	0805000	S9B	S9	α/β-hydrolase	1225600 / dispensable	low fitness	dispensable in asexual stages[j]	ES	G	S, G
	0321500	S9A		acylaminoacyl-peptidase	1217000	dispensable	dispensable in asexual stages[j]	ES[f]	S, Sp	S, G
	1126600			steryl-ester hydrolase	0921800 / dispensable	dispensable		[f]	G	T, G
	0818600	PBLP	BEM46-like	α/β-hydrolase	0712200 / dispensable	essential	merozoite defect in asexual and liver stages in *P. yoelii*[k]	ES[f]	S, G, Sp, Oc	S, G, Sp, Oc
	1129300			α/β-hydrolase	0918900 / dispensable	dispensable		ES, MS[f]	S, Sp	S, Sp
	1134500		PGAP1-like	α/β-hydrolase	0913900	essential		ES[f]	M, G, Sp	T
	1143000	abH114		α/β-hydrolase	0906000 / dispensable	essential	dispensable in asexual stages	ES[f]	S, G, Sp	S, G, Sp, Oc
	1116100			serine esterase	0931900 / dispensable	dispensable		ES	S, M, Sp	S, Sp
	0630100			α/β-hydrolase	1128900 / dispensable	dispensable				R, S, Sp, Ok
	0814400			phospholipase DDHD1	1423100 / slow	essential			S, Sp	S, Sp, G, Ok
	1306200			α/β-hydrolase	1404700 / essential	essential			R, T, S, G, Sp, Oc	S, Oc
	1458300			α/β-hydrolase	1322000 / dispensable	dispensable		ES[f]	Sp	Sp, S
	0808000			α/β-hydrolase	1222700 / slow	essential				T, Oc, Sp
PLPs	0209100	PL1	Patatin-like	phospholipase A2	0306200	dispensable		M[f]	S, G	T, Oc
	0924000		Patatin-like	Phospholipase	0824900 / dispensable	dispensable			S, G	S, Sp
	1358000		Patatin-like	Phospholipase	1134300 / essential	essential		ES[f]	S, G, Sp	S, Oc
	0218600		Patatin-like	Phospholipase	0315300	dispensable		T, ES[f]	S, M	T, Oc
	0416100	GATA	Amidase	glutamyl-tRNA(Gln) amidotransferase A	0718100 / slow	essential	essential in asexual stage *P. berghei*[l]			S, Sp

[a](Schwach et al., 2015).
[b](Zhang et al., 2018).
[c](Aurrecoechea et al., 2009; Florens et al., 2002; Lasonder et al., 2015; Oehring et al., 2012; Silvestrini et al., 2010).
[d](Aurrecoechea et al., 2009; Gómez-Díaz et al., 2017; López-Barragán et al., 2011; Otto et al., 2010; Pelle et al., 2015; Toenhake et al., 2018; Zanghì et al., 2018).
[e](Istvan et al., 2017).
[f]Identified in FP-Biotin schizont screen (Elahi et al., 2019b). G, gametocyte; ES, early schizonts; M, merozoites; Oc, oocyst ;Ok, ookinetes; R, rings; S, schizont; Sp, sporozoites; T, trophozoites.
[g](Burda et al., 2015).
[h]Dr Natalie Spillman, personal communication.
[i](Spillman et al., 2016).
[j]Dr S. Ridewood, personal communication.
[k](Groat-Carmona et al., 2015).
[l](Mailu et al., 2015).

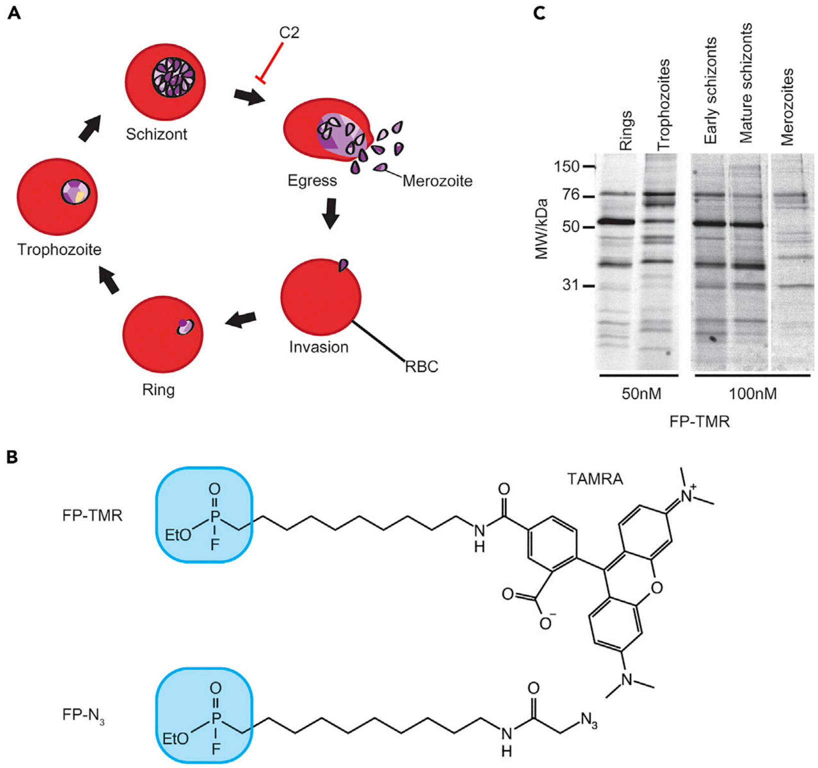

Figure 1. SHs are differently expressed or activated throughout the erythrocytic cycle

(A) The *Plasmodium falciparum* asexual life cycle. C2 treatment arrests parasites 15 min before egress.

(B) Structure of FP ABPs probes used in this study containing either a TAMRA fluorophore or an azide group for tandem-tagging via CuAAC chemistry. The fluorophosphonate warhead is shown in blue.

(C) Parasite lysates collected at the indicated life stages were labeled for 30 min with 50 or 100 nM FP-TMR. After SDS-PAGE, in-gel fluorescence was measured using a fluorescence scanner. Each band in the lane likely correspond to a different active SH.

RESULTS

ABPP of SHs across the erythrocytic cycle using a fluorescent FP probe

Merozoites, rings, trophozoites, and schizonts were collected from synchronous cultures of *P. falciparum* 3D7 parasites. Merozoites were purified from a culture of actively rupturing schizonts by centrifugation. Ring, trophozoite, and schizont stage parasite pellets were collected after saponin treatment, which lyses the RBC and PV membranes. This removes the majority of hemoglobin, which interferes with in-gel fluorescence readout and proteomics sample preparation, but also proteins exported into the PV or RBC. Schizonts were purified at two different stages: early schizonts, during which nuclear division is taking place but cytokinesis has not yet occurred; and mature schizonts, where merozoites have been fully formed. To obtain the latter stage, purified mature schizonts were cultured for 4h with the reversible PKG inhibitor Compound 2 (C2). C2 arrests parasite development 15 min before egress (Collins et al., 2013b).

Soluble protein lysate from each life stage was labeled with 50–100 nM of FP-TMR (Figure 1B) for 1h at RT. Samples were run on SDS-PAGE gels and in-gel fluorescence measured with a fluorescence scanner. Across all life stages, approximately 22 different proteins were labeled, and a different activity pattern was seen at each stage (Figure 1C), indicating cyclic expression and/or activation of SHs. Therefore, to identify and profile as many *Pf*SHs as possible we performed chemical proteomics experiments at different stages of development.

ABPP of SHs by quantitative chemical proteomics

The protocol to accurately profile and quantify stage-dependent SHs activities is represented in Figure 2. Intact parasites at ring (0–20 hpi), trophozoite (20–36 hpi) and early (36–44 hpi) or mature

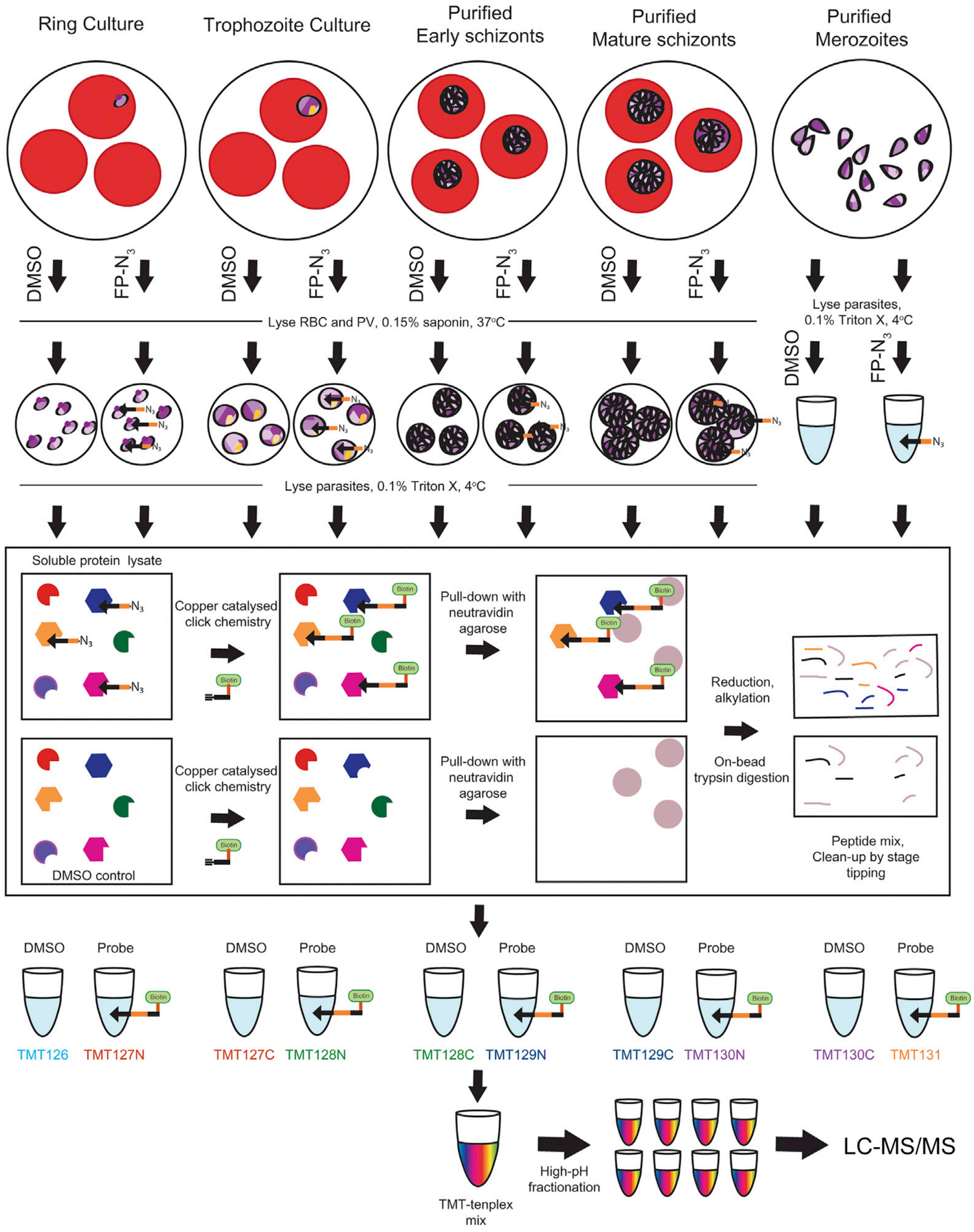

Figure 2. ABPP protocol for profiling SHs throughout the erythrocytic cycle

Ring, trophozoite and early or late schizont stage parasites were treated with 1 μM FP-N_3 or DMSO under intact conditions. Parasites were then washed, saponin permeabilized and frozen. Merozoites were lysed before probe labeling. The soluble protein was then extracted, and CuAAC-chemistry used to attach biotin to labeled proteins for pull-down on neutravidin agarose beads. After on-bead reduction and alkylation of proteins, trypsin digestion liberates peptides for subsequent processing for proteomics. Peptide samples are labeled with different 10-plex TMT tags and combined before high pH fractionation and LC-MS/MS analysis. See also Figure S1.

(48 hpi, pre-egress) schizont stages were treated with 1 μM FP-N_3 or DMSO for 1h. Intact cells were then washed to remove excess unreacted probe before saponin treatment. Parasite pellets were washed multiple times with PBS to remove excess hemoglobin and frozen in liquid N_2. Merozoites (48 hpi, post-egress) have a very short lifespan outside the RBC and are difficult to collect in large quantities. Therefore, they were collected and frozen before probe (1 μM FP-N_3) or DMSO treatment in lysates for 1h.

CellPress
OPEN ACCESS

For each life stage and treatment, triton X-soluble protein fractions were extracted and pooled. Samples of equal protein content were split into three technical replicates and treated with CuAAC-chemistry reagents to conjugate an alkyne-biotin tag to FP-N_3 modified proteins. Labeled proteins were affinity purified using a 10:20 μL mixture of neutravidin:blank agarose beads. These conditions were chosen to optimize depth of coverage (Figure S1). Captured proteins were reduced, alkylated and trypsin digested on-bead, and the eluted peptides cleaned up by stage tipping. Peptides obtained from each of the ten different conditions (five life stages treated with FP-N_3 or DMSO) were labeled with different TMT tags and combined. Each of the triplicate 10-plex TMT mixes was split into 8 fractions by high-pH fractionation before LC-MS/MS analysis to decrease sample complexity and increase depth of coverage.

1024 proteins were identified by MaxQuant analysis of the LCMS/MS raw data. Proteins were filtered to keep only proteins identified from more than 4 peptides in each of the 3 replicates and in at least one life stage, giving a final list of 405 proteins. The $\log_2$ intensities were normalized by subtracting the median intensities of each replicate. Thirty-five proteins were significantly enriched in probe vs DMSO treated samples in at least one life stage (Figure 3A, Student's t-test, FDR = 0.05, s_0 = 0.5; Table S1): 27 *P. falciparum* proteins and 8 *Hs*SHs that are discussed below.

Of the 27 enriched *P. falciparum* proteins, only two are not predicted to be SHs: a proteasome proteolytic subunit (beta type-5) and an endonuclease (MUS81). Proteasome subunits have been previously identified with FP probes (Jessani et al., 2005), but the interaction with MUS81 is unknown. Of the 25 *Pf*SHs identified, three were PLPs and the rest *Pf*α/βHs including predicted lysophospholipases, lipases, and peptidases (Table 1). We were surprised to detect PfXL2, a non-essential exported lipase, which we expected to be lost after saponin treatment (Spillman et al., 2016). However, the related PfXL1 was not detected. Among the 25 *Pf*SHs identified here, eight were unique to this study compared to the previous one where they use the FP-desthiobiotin probe in schizonts lysates (Elahi et al., 2019b). Two of these, PF3D7_1476700 (Psta1) and PF3D7_1427100, demonstrate the highest activity in merozoite and trophozoite stages respectively, which might explain their absence from the FP-Biotin study. The remaining six are active in schizont stage but may be less active in lysates or might not have been detected because of differences in sample preparation. On the other hand, the previous study identified three unique *Pf*SHs (Elahi et al., 2019b): PF3D7_1126600, predicted to be a steryl ester hydrolase; *Pf*EH2, which is associated with the RBC periphery (Spillman et al., 2016); and the rhomboid protease ROM4, involved in the shedding of merozoite surface proteins during RBC invasion. These three SHs were detected in the insoluble fraction of schizont lysates and might be strongly associated with membranes. Indeed, ROM4 has seven transmembrane domains. Therefore, our lysis/solubilization conditions might not have been strong enough to extract these SHs.

To quantify changes in SHs activities during the asexual cycle, the average $\log_2$ enrichment at each life stage was used to generate a heat map of hydrolase activity over time (Figure 3A). SHs were grouped by Euclidean clustering based on their temporal profile. The majority of *Pf*SHs had 4- to 64-fold changes in activity between life stages, with the lowest level of activity usually at ring stage. The dynamic temporal activation of *Pf*SHs suggests they may play stage-specific roles in metabolism. PF3D7_1129300, PF3D7_1476800, PF3D7_0629300 (*Pf*LCAT) and PF3D7_1134500 seem to be most active at schizont stages, and PF3D7_1458300, PF3D7_1358000 and PF3D7_1120400 (abH112) in early schizonts. The activities of Psta1, PF3D7_1328500 (MLPL), and RhoSH increase significantly between mature schizonts and merozoites. We hypothesized that changes in activity between these two stages may represent an activation event essential to egress or invasion.

Genetic interrogation of *Pf*SHs by conditional allelic replacement

Four SHs of unknown function were selected for genetic interrogation. Of the SHs active at merozoite stage, MLPL was annotated as dispensable in a genome-wide *piggyBac* transposon screen (Zhang et al., 2018) and was not pursued in this study. RhoSH was annotated as important and was already under investigation in our lab (Dr S. Ridewood, personal communication). Psta1, part of the *Plasmodium* sub-telomeric a family (Carlton et al., 2002; Templeton, 2009), was not annotated as essential on the *piggyBac* screen, but its disruption resulted in a strong fitness cost. Psta1 was chosen for genetic interrogation with the close paralogue Psta2 (PF3D7_1476800), which is most active at schizont stage and was annotated as essential.

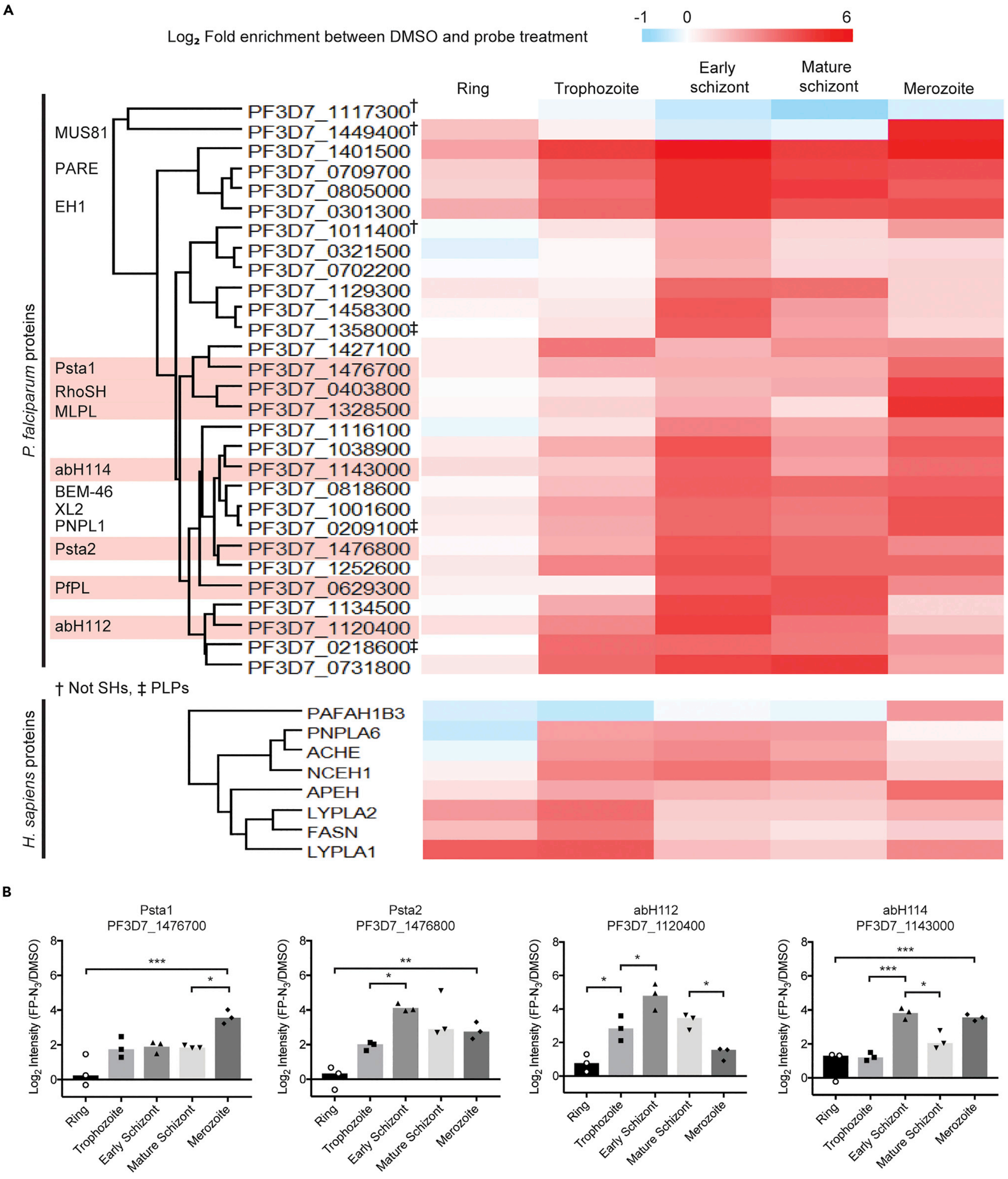

Figure 3. Activity profile of SHs throughout the erythrocytic cycle

(A) For each protein, fold enrichment was calculated as Log_2(Intensity(FP-N_3/DMSO)) and used to create a heat map. Proteins were clustered by Euclidean clustering. Proteins discussed in the text are highlighted. † denotes non-SHs, ‡ denotes SHs of PLP-type, all others are α/β-fold hydrolases.

(B) The Log_2 enrichment of four selected PfSHs for genetic interrogation are plotted against life cycle stage. The bars represent the mean of three replicates (replicates shown by symbols). Statistical significance was determined by unpaired one-way ANOVA. Significance levels are indicated: $p \leq 0.001$, ***; $p \leq 0.01$, **; $p \leq 0.05$, *; $p < 0.05$, non-significant. See also Table S1.

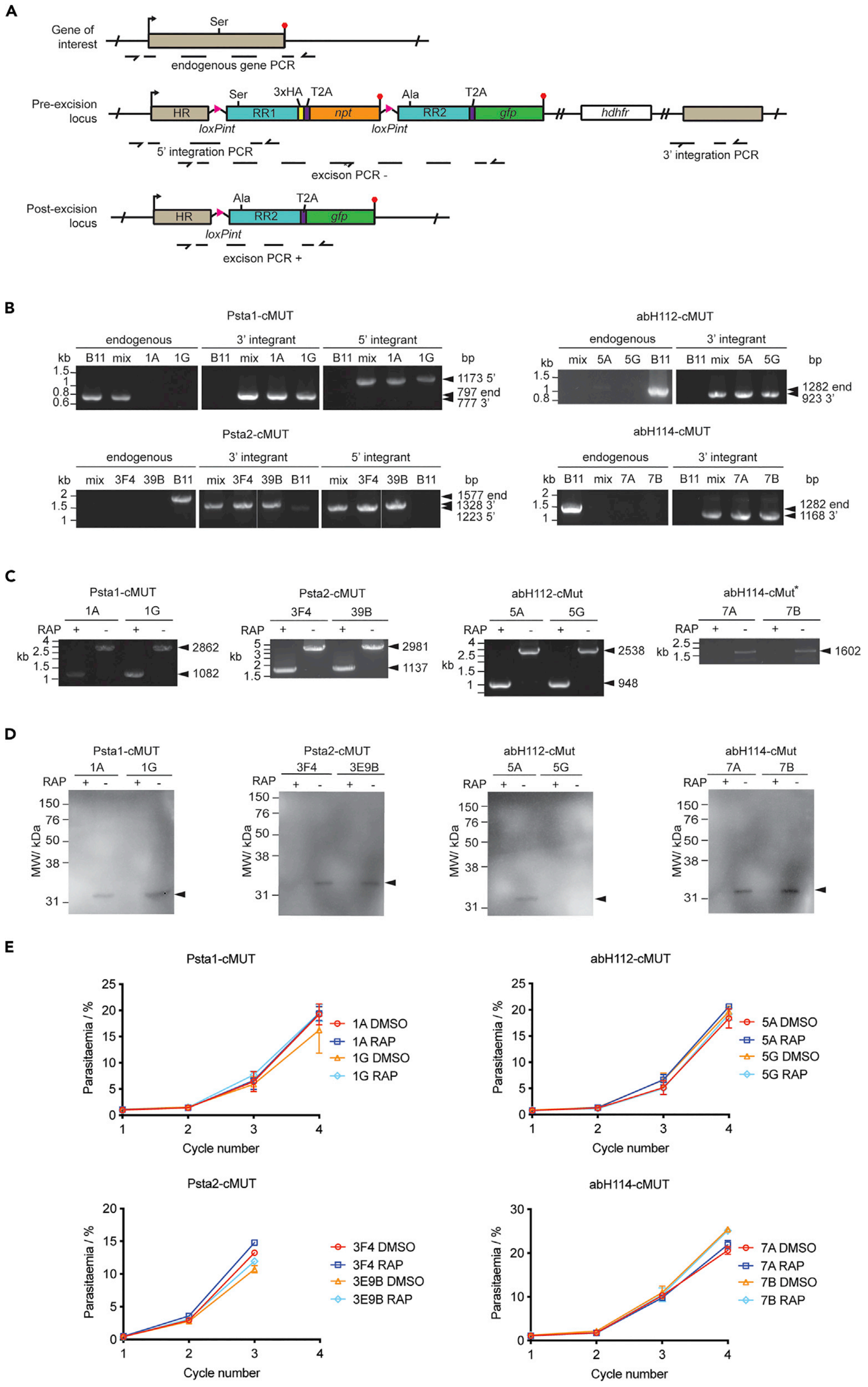
A
Gene of interest
Ser
endogenous gene PCR
Pre-excision locus
HR
Ser
RR1
3xHA
T2A
npt
loxPint
Ala
RR2
T2A
gfp
hdhfr
5' integration PCR
excison PCR -
3' integration PCR
Post-excision locus
HR
Ala
RR2
T2A
gfp
loxPint
excison PCR +
B
Psta1-cMUT
endogenous
3' integrant
5' integrant
kb
B11 mix 1A 1G
B11 mix 1A 1G
B11 mix 1A 1G
bp
1.5
1
0.8
0.6
1173 5'
797 end
777 3'
abH112-cMUT
endogenous
3' integrant
mix 5A 5G B11
B11 mix 5A 5G
1282 end
923 3'
Psta2-cMUT
endogenous
3' integrant
5' integrant
mix 3F4 39B B11
mix 3F4 39B B11
mix 3F4 39B B11
2
1.5
1
1577 end
1328 3'
1223 5'
abH114-cMUT
endogenous
3' integrant
B11 mix 7A 7B
B11 mix 7A 7B
1282 end
1168 3'
C
Psta1-cMUT
1A
1G
RAP
2862
1082
Psta2-cMUT
3F4
39B
2981
1137
abH112-cMut
5A
5G
2538
948
abH114-cMut*
7A
7B
1602
D
Psta1-cMUT
1A
1G
RAP
MW/ kDa
150
76
50
38
31
Psta2-cMUT
3F4
3E9B
abH112-cMut
5A
5G
abH114-cMut
7A
7B
E
Psta1-cMUT
Parasitaemia / %
Cycle number
1A DMSO
1A RAP
1G DMSO
1G RAP
abH112-cMUT
5A DMSO
5A RAP
5G DMSO
5G RAP
Psta2-cMUT
3F4 DMSO
3F4 RAP
3E9B DMSO
3E9B RAP
abH114-cMUT
7A DMSO
7A RAP
7B DMSO
7B RAP

Figure 4. Genetic interrogation *of P. falciparum* SHs

(A) Conditional allelic replacement strategy. The p2TA-cMUT construct has a homology region (HR, grey), loxPint sites (pink triangles), two differentially recodonized regions (RR1& RR2, turquoise), a triple HA tag (yellow), a T2A peptide sequence (purple), a *npt* selection marker (orange), *gfp* (green) and a *hdhfr* cassette (white). Upon RAP treatment, the sequence between the two loxPint sites is excised (post-excision locus). Arrows and red hexagons indicate start and stop codons, respectively. Primer binding sites are shown with dashed lines and half arrows.

(B) Diagnostic integration PCRs. Primers were design to detect the endogenous locus or the 5′ and 3′ end integration sites. PCRs were performed on gDNA from pre-clonal (mix) and 2 clonal lines. B11 gDNA was used as a negative control. For abH112-cMUT and abH114-cMUT PCR conditions to observe 5′ integration could not be obtained.

(C) Excision PCRs were performed on gDNA from DMSO- or RAP-treated schizonts or merozoites. RAP treatment results in the smaller PCR products or lack of it.

(B and C) Primer binding sites are shown in A. The expected sizes of the PCR products are shown by arrowheads.

(D) WB analysis for two clones per cMUT line was performed on lysates from DMSO- or RAP-treated schizonts or merozoites using an anti-HA antibody. RAP treatment results in the loss of HA signal.

(E)Parasite replication assay of DMSO- or RAP-treated parasites from two clonal lines per cMUT was carried out under standard conditions starting at 0.1% parasitemia. Samples were taken at 45 hpi in the first cycle, and approximately at 30 hpi in each subsequent cycle. Parasites were fixed, stained with Hoechst, and parasitemia quantified by flow cytometry. Two clones are represented in black and grey, DMSO and RAP treated samples by filled and empty circles, respectively. Data is represented as mean +/− standard deviation of three replicates.

(B–E) cMUT clones: Psta1, 1A & 1G; Psta2, 3F4 & 3E9B; abH112, 5A & 5G; abH114, 7A & 7G.

In addition, we selected two *Pf*SHs of unknown function that have also been annotated as essential: abH112 and abH114 (PF3D7_1143000). abH112 activity peaks in early schizont stage, and abH114 in early schizont and merozoite stages (Figure 3B). Only abH114 has an ortholog in *Plasmodium berghei*, PBANKA_0906000, which was annotated as dispensable based on the PlasmoGEM genetic screen (Schwach et al., 2015). However, there are examples of differences in lipid metabolism pathways between rodent and human malarial parasites (Déchamps et al., 2010a,2010b). abH112 is conserved among human-infecting parasites, and Psta1 and Psta2 are conserved in other *Plasmodium laverania* sub species, such as *Plasmodium reichenowi* and *Plasmodium gaboni*. Large-scale genetic screens are a very useful resource; they can be combined with complementary molecular biology techniques such as conditional KO (cKO) to validate gene essentiality.

cKO allows us to monitor the downstream effects of gene loss in real-time. To specifically determine whether the activity of selected SHs is essential, we designed a strategy to conditionally mutate the catalytic Ser to Ala which combines DiCre, artificial introns containing a *LoxP* site (LoxPint; Matera and Wang, 2014; Pieperhoff et al., 2014), and selection-linked integration (SLI). The DiCre system uses Cre recombinase constitutively expressed as two domains fused to rapamycin (RAP) binding domains. RAP treatment leads to dimerization and activation of DiCre and subsequent rapid excision of LoxP flanked DNA sequences (Collins et al., 2013a; Jullien et al., 2003). Our constructs contain a human dihydrofolate reductase (*hdhfr*) cassette to select for transfected parasites using the drug WR99210 (WR). In SLI, a neomycin phosphotransferase gene (*npt*) confers resistance to the drug G418. The *npt* is placed downstream of the modified gene of interest (GOI) via a viral ribosome skipping T2A peptide (Kim et al., 2011). After plasmid integration by single homologous recombination, the GOI and *npt* are translated as two proteins from a single mRNA, thus allowing us to select for integration (Birnbaum et al., 2017).

Our constructs (pT2A-cMUT) contain an N-terminal homology region (HR) to target the construct to the GOI. The rest of the recodonized gene (including the wild-type catalytic region, RR1), a C-terminal triple HA tag, the T2A coding sequence and *npt* are flanked with LoxPint sequences. This is followed by a second version of the recodonized region (RR2) containing an Ala codon in place of the catalytic Ser. RR2 is fused to *gfp* via a T2A peptide sequence. After transfection and selection of WR resistant parasites (1–2 weeks), G418 is added to select for parasites that have integrated the plasmid. RAP treatment of G418-resistant parasites induces excision of LoxPint flanked DNA sequences, resulting in loss of *npt*, replacement of the RR1 by RR2, and expression of a Ser to Ala SH mutant (SH-cMUT, Figure 4A) and free GFP.

Schizonts purified from a culture of *P. falciparum* DiCre-expressing B11 parasites (Perrin et al., 2018) were transfected with pT2A-cMUT constructs designed for each GOI (Figure 4A). Four parasite lines were generated (Psta1-cMUT, Psta2-cMUT, abH112-cMUT and abH114-cMUT), and two clones selected for each one. Integration in pre-clonal and clonal lines was assessed by PCRs to detect the endogenous gene or 3′ and 5′ integration sites (Figure 4A). Integration of the constructs into *Psta2*, *abH112* and *abH114* loci was highly

efficient, as shown by the absence of an endogenous PCR product in the pre-clonal population (Figure 4B). However, an endogenous PCR product was observed for pre-clonal Psta1 (Figure 4B). 5′ integration PCRs were never obtained for abH112 or abH114. Because all subsequent experiments supported integration, it was assumed that this was due to unyielding chromatin structure.

To test the excision efficiency of each cMUT line, synchronous parasite cultures were treated for 3h with DMSO or RAP at ring stage. Parasite samples were collected at schizont (Psta2-cMUT, abH112-cMUT, abH114-cMUT) or merozoite (abH112-cMUT) stages to extract genomic DNA (gNDA) and prepare protein lysates. As expected, RAP treatment resulted in smaller PCR product for Psta1, Psta2 and abH112, or a lack of it for abH114 (Figure 4C). These results demonstrate efficient excision in all lines. Western blot (WB) analysis of parasite lysates confirmed the loss of the HA-tagged SHs after excision for all lines, except for one abH112 clone (5G) for which no HA signal was detected (Figure 4D).

After validation of excision efficiency at the DNA and protein level, we looked at the effect of conditional mutation of the catalytic Ser on parasite replication. Synchronous ring-stage parasites from each cMUT line were treated with DMSO or RAP at ring stage. After 12h, RAP was removed, and cultures were transferred to 96-well plates (0.5 hematocrit at 0.1% parasitemia). Samples were collected every 48h, and parasitemia quantified by flow cytometry over 3 to 4 cycles (Figure 4E). None of the allelic replacement mutants showed a significant decrease in parasite replication, indicating that the catalytic activities of Psta1, Psta2, abH112 and abH114 are not important for parasite development in culture. This is in contradiction to the large-scale genetic screening results (Zhang et al., 2018). However, we cannot rule out that these proteins might play an important non-catalytic function in *P. falciparum*.

Human SHs may be potential antimalarial targets

Upon RBC invasion, parasites remodel the RBC, and some host enzymes and pathways are co-opted. Exploring the interaction between parasite and host SHs could provide a new source of antimalarial targets. We identified 8 active *Hs*SHs in iRBCs (Figures 3A and Table 2). Although saponin permeabilization should preclude the detection of *Hs*SHs, some RBC membrane debris could remain in the parasite pellet. This could explain the presence of membrane associated SHs such as acetylcholine esterase (AChE, Ott, 1985). Alternatively, cytosolic *Hs*SHs could be imported into the parasite as has been reported for other RBC metabolic enzymes such as ALAD and hPrx-2 (Bonday et al., 2000; Koncarevic et al., 2009). The RBC performs no *de novo* protein synthesis. Differences in *Hs*SHs activity could reflect degradation of active enzyme, or a change in activation state or localization. All identified *Hs*SHs showed some life-stage-dependent change in active enzyme abundance (Figure 3A). Platelet activating factor acetyl hydrolase 1B gamma (PAFAH1B3) and acyl-amino acid releasing enzyme (APEH) were most activate at merozoite stage, the acyl protein thioesterases (LYPLA1 and LYPLA2) and fatty acid synthase (FASN) at ring and trophozoite stages, and AChE, neutral cholesterol esterase (NCEH1) and neuropathy target esterase (PNPLA6) at trophozoite and schizont stages. These results suggest that the enzymes may have stage-specific roles to play in parasite growth and replication. Many of these *Hs*SHs have been extensively characterized, and inhibitors are commercially available (Table 2 and Figure 5A). We therefore purchased eight inhibitors to measure their antiparasitic activity.

Five of the inhibitors tested have been shown to be highly selective in mammalian systems using FP probe-based ABPP studies: P11 selectively and reversibly inhibits PAFAH1B3 and the related PAFAH1B2, which was not identified in this study (Chang et al., 2015); ML-348 and ML-349 selectively and reversibly inhibit LYPLA1 and LYPLA2, respectively (Adibekian et al., 2010a, 2010b, 2012); JW480 is a highly potent and selective reversible inhibitor of NCEH1 (Chang et al., 2011); and AA74-1 selectively inhibits APEH (Adibekian et al., 2011). TVB-3166 reversibly inhibits FASN via the keto-reductase unit (Huesca et al., 2005). This disrupts palmitate synthesis *in vivo* and on-target selectively was demonstrated by rescue with exogenous palmitate (Heuer et al., 2016; Ventura et al., 2015). TOCP is an organo-phosphophosphate (OP). OPs are responsible for human OP neuropathy, caused by inhibition of *Hs*SHs in the brain. TOCP has been shown to be selective for PNPLA6 over AChE (Johnson, 1969, 1975; Veronesi et al., 1991). The carbamate pyridostigmine bromide (PyBr) can protect from AChE-dependent OP poisoning by reversible inhibition of AChE and has been shown to target the erythrocyte variant (Dawson, 1994; Herkert et al., 2011; Koster, 1946). Though their primary human targets are known, TOCP and PyBr have the least evidence of selectivity. Despite the reported on-target specificities of all inhibitors in mammalian systems, their selectivity in *P. falciparum* is unknown.

Table 2. Human SHs and their inhibitors

Identifier	Name	Biochemical Activity	Biological Function	Inhibitor	IC_{50} (nM)	*Pf*EC_{50} (µM)
PAFAH1B3	Platelet activating factor acetyl hydrolase 1B gamma	Phospholipase A2 activity	Hydrolyses an acetyl group from the glycerol backbone of platelet-activating factor and circulating aspirin in erythrocytes (Hattori and Arai, 2015; Kohnz et al., 2015).	P11 (Chang et al., 2015) Reversible	880	–
FASN	Fatty acid synthase	Multi-domain fatty acid synthase (Chang and Hammes, 1990)	The malonyl-CoA-/acetyl-CoA-ACP-transacylase (MAT) and thioesterase (TE) domains use nucleophilic serines (Bunkoczi et al., 2009; Pemble et al., 2007).	TBV-3166 (Heuer et al., 2016) Reversible	42 (Ventura et al., 2015)	14 ± 5
PNPLA6	Neuropathy target esterase	Patatin-like phospholipase (Kienesberger et al., 2009)	Membrane associated (Tienhoven et al., 2002). Plays a critical role in the mammalian brain via hydrolysis of phosphotidyl choline (Quistad et al., 2003; Richardson et al., 2013).	TOCP (Davis and Richardson, 1980; Richardson et al., 2013) Irreversible	150 (Veronesi et al., 1991)	7 ± 1
LYPLA1 (APT1)	Acyl protein thioesterase	Depalmitoylase (Duncan and Gilman, 1998)/ lysophospholipases (Sugimoto et al., 1996)	APT1/APT2 share 60% identity and some substrates.	ML348 (Won et al., 2016) Reversible	840 (Adibekian et al., 2010a)	1.7 ± 0.2
LYPLA2 (APT2)	Acyl protein thioesterase			ML349 (Won et al., 2016) Reversible	510 (Adibekian et al., 2010b)	–
AChE	Acetylchloline esterase	Hydrolysis of acetylcholine (Ach)	GPI anchored to the plasma membrane in erythrocytes (Ott, 1985). Possible non-esterase, membrane scaffolding activities (Soreq and Seidman, 2001). Nitric oxide signal transduction (Saldanha, 2017).	Pyridostigmine bromide (PyBr) (Herkert et al., 2011) Reversible	330	~12
NCEH1 (KIAA1363/ AADACL1)	Neutral cholesterol esterase	Esterase	Membrane glycoprotein, hydrolysis of cholesterol esters (Sekiya et al., 2011) and 2-acetyl-monoalkylglycerol ethers (Buchebner et al., 2010; Chiang et al., 2006).	JW480 (Chang et al., 2011) Irreversible	12	–
APEH	Acyl-amino acid releasing enzyme	Hydrolyses acyl-amino acids from peptides (Tsunasawa et al., 1975)	Adherent to oxidized erythrocyte membranes and preferentially degrades oxidatively damaged proteins (Fujino et al., 2000).	AA74-1 (Adibekian et al., 2011) Irreversible	5 (Adibekian et al., 2011)	0.20 ± 0.01

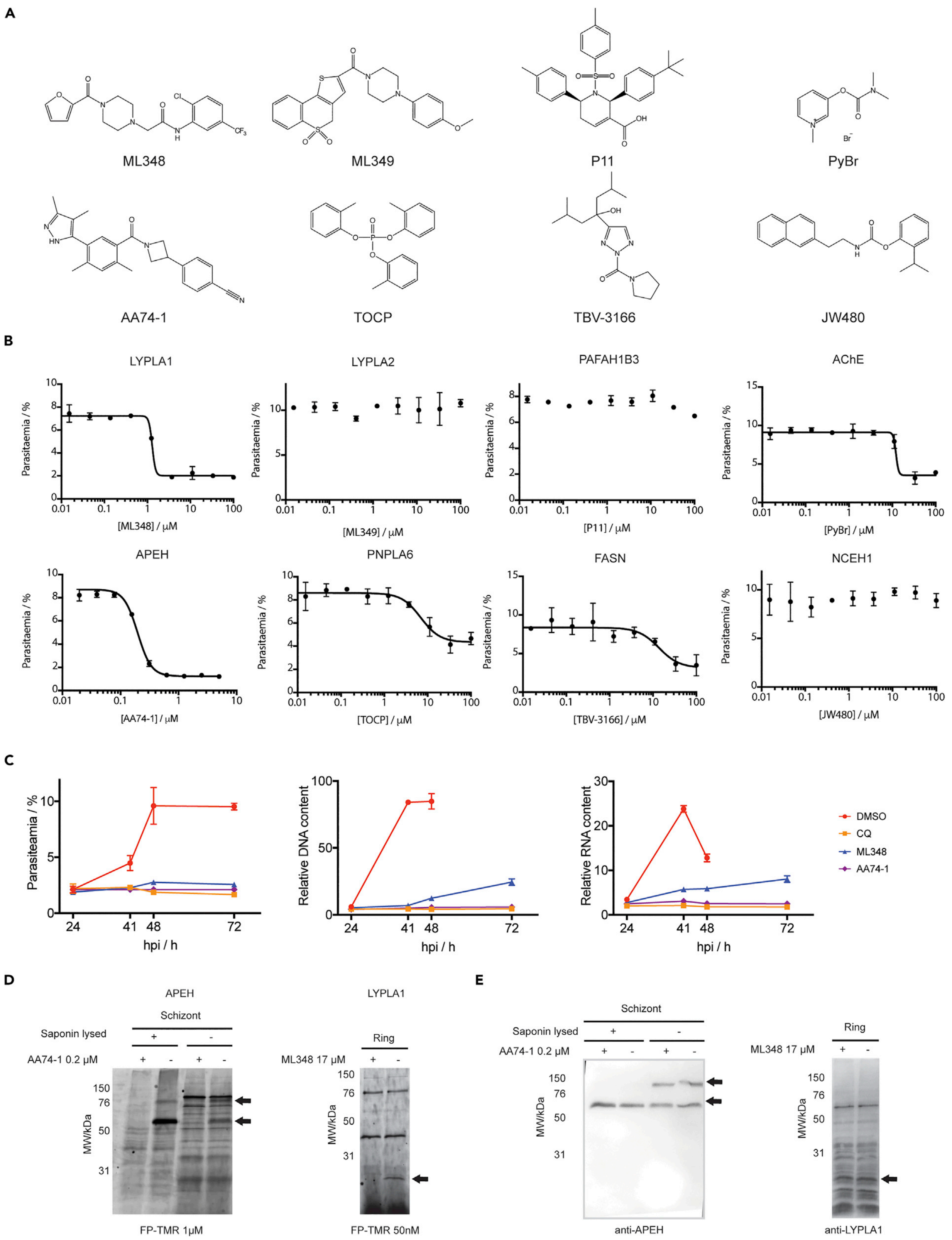
A
ML348
ML349
P11
PyBr
AA74-1
TOCP
TBV-3166
JW480
B
LYPLA1
LYPLA2
PAFAH1B3
AChE
APEH
PNPLA6
FASN
NCEH1
Parasitaemia / %
[ML348] / μM
[ML349] / μM
[P11] / μM
[PyBr] / μM
[AA74-1] / μM
[TOCP] / μM
[TBV-3166] / μM
[JW480] / μM
C
Parasiteamia / %
Relative DNA content
Relative RNA content
hpi / h
DMSO
CQ
ML348
AA74-1
D
APEH
Schizont
Saponin lysed
AA74-1 0.2 μM
MW/kDa
FP-TMR 1μM
LYPLA1
Ring
ML348 17 μM
FP-TMR 50nM
E
Schizont
Saponin lysed
AA74-1 0.2 μM
anti-APEH
Ring
ML348 17 μM
anti-LYPLA1

Figure 5. Investigation of *Hs*SHs as potential antimalarial targets

(A) Structures of HsSH inhibitors.

(B) Standard 72h replication assay was carried out in the presence of different concentrations of *Hs*SH inhibitors. Parasites were fixed, stained with Hoechst, and parasitemia quantified by flow cytometry. Data is represented as mean +/- SEM of three replicates and was fitted to a dose response curve to determine EC_{50} values (Table 2).

(C) Ring stage parasites were treated with DMSO, CQ or 3 times the EC_{50} value concentration measured for ML348 or AA74-1. Cultures were fixed 24, 41, 48 and 72 h after treatment, DNA and RNA stained with Hoechst and 132A respectively, and analyzed by flow cytometry. Parasitemia was calculated at each time point, with median DNA and RNA signal normalized to the background signal of uRBCs. For samples in which two distinct iRBCs populations (schizonts and rings) were observed at the time of egress and invasion, only the RNA and DNA level of the schizont population is shown in the graph. These correspond to DMSO treatment at 41 and 48 hpi, and ML348 treatment at 72 hpi (B and C). Data is represented as mean $\pm$ SEM of three replicates.

(D) Schizont (whole or saponin-treated) and ring (saponin-treated) lysates were pre-treated with 0.2 μM ML348 or 17 μM AA74-1 before being labeled with 1 μM or 50nM FP-TMR. After SDS-PAGE, in-gel fluorescence was measured using a fluorescence scanner. Missing bands corresponding to inhibited SHs are indicated with arrows.

(E) WB analysis of gels from (D) was performed using monoclonal anti-APEH or anti-LYPLA1 antibodies. Bands of interest are indicated with arrows. See also Figure S2.

Ring-stage parasites were treated with DMSO or different concentrations of inhibitor for 72h, and parasitemia was measured by flow cytometry. The LYPLA1, APEH, PNPLA6, FASN, and AChE inhibitors were all found to affect parasite replication in a dose dependent manner, although full dose response curves were not obtained for FASN and AChE (Figure 5B). The inhibitors of the other three *Hs*SHs had no effect in parasite replication, including the LYPLA2 inhibitor ML349 (Figure 5B). This was surprising because LYPLA1 and LYPLA2 have been shown to have the same biochemical function and share 60% identity (Long and Cravatt, 2011). These results support our hypothesis that some *Hs*SHs may be co-opted by the parasite and are essential for growth and replication. We further interrogated the inhibition of APEH and LYPLA1 because their inhibitors showed the lowest EC_{50} values and completely blocked parasite replication at high concentrations (Table 2 and Figure 5B).

To better understand the effect of LYPLA1 and APEH inhibitors in parasite development, we used a newly developed flow cytometry assay able to monitor the *P.* falciparum erythrocytic cycle with high temporal resolution (Bell et al., 2021). In this assay, fixed parasite cultures are stained with selective DNA (Hoechst) and RNA (132A) dyes (Cervantes et al., 2009). By monitoring the level of DNA and RNA in iRBCs we can track a synchronous parasite culture throughout the life cycle and distinguish between uRBCs, low background staining signals; rings, low DNA and RNA content; trophozoites, low DNA, medium RNA content; and schizonts, high DNA and RNA content (Figure S2). Thus, using this assay we can determine the stage at which inhibitors arrest parasite development.

Synchronous parasites were treated immediately after RBC invasion (1 hpi) with 5 μM ML348 or 0.625 μM AA71-1 (3 × EC_{50} value, Table 2). DMSO was used as a negative control, and chloroquine (CQ) as a positive control that kills parasites immediately after treatment. Samples were collected and fixed at 24, 41, 48 and 72 hpi. Total parasitemia and the median DNA and RNA content of treatment cycle parasites was measured by flow cytometry (Figure 5C).

Parasites treated with DMSO developed as expected, i.e. increase in RNA/DNA signals between 24 and 48 hpi and an increase in parasitemia reflected by a large population of rings with low RNA/DNA levels, whose RNA signal increases in trophozoite stage at 72 hpi (Figures 5C and S2). None of the small molecules used affected uRBC morphology (Figure S2). CQ-treated samples did not progress indicating ring-stage death (Figures 5C and S2). ML348-treated parasites appeared to slow their development throughout the life cycle. At 48 hpi, iRBCs show significantly lower DNA and RNA levels compared to DMSO controls (Figure 5C). Parasitemia increased slightly, indicating that ML348 significantly delays schizogony but a small proportion of parasites are able to progress to the next cycle at 5 μM ML348 (Figure S2). Our ABPP results indicated that LYPLA1 is most active at ring and trophozoite stages (Figure 3A). Taken together, these data suggest that if LYPLA1 is the main target of ML438, its metabolic function in trophozoites is likely important for parasite to successfully undergo schizogony.

AA74-1-treated parasites appeared to arrest development early in the cycle similarly to CQ-treated ones (Figure 5C). Indeed, no significant increase in DNA and RNA levels was observed between the 24 hpi and the later time points. This also indicates that AA74-1 is a very fast acting compound. Our ABPP results indicate that APEH is most active in merozoites (Figure 3A), suggesting that it is internalized in parasites and that it has a role immediately after invasion.

CellPress
OPEN ACCESS

In order to assess the selectivity of the *Hs*SH inhibitors, we tested them in competition with FP-TMR in parasite lysates. In schizonts, pre-treatment with AA74-1 inhibited FP-TMR labeling of two bands corresponding to full-length and processed APEH as previously described (Figures 5D and 5E, Elahi et al., 2019a). When schizonts were saponin-treated before the competition assay, only the lower MW band was observed, suggesting that only the processed form of this protein is internalized in the parasite. These data support the findings of a recent article that showed evidence for APEH internalization and its essential role in *P. falciparum* asexual replication (Elahi et al., 2019). In ring lysates, competition of the reversible inhibitor ML348 with sub-saturating concentration of FP-TMR prevented labeling of a single band at the expected molecular weight for LYPLA1 (25kDa, Figure 5D). A band was observed at the same MW by WB, but there was too much non-specific binding to be confident that this is LYPLA1 (Figure 5E). Although these data point to the specificity of AA74–1 and ML348 for APEH and LYPLA1 respectively, there may be low abundant or lowly/unlabeled targets that are not identified by competition ABPP. To test if *Pf*SH targets are involved in AA74–1 or ML348-dependent growth inhibition, a chemical proteomics strategy could be employed similar to techniques recently used to find targets of Salinipostin A (Yoo et al., 2020).

DISCUSSION

In recent years, the need for new antimalarial treatments with novel modes of action has led to a refocus on target-based drug discovery in the field. Metabolic SHs play essential functions in many biochemical processes, such as lipid metabolism and posttranslational modification. SHs have been proven to be druggable, but they remain unexplored as a source of potential antimalarial targets. In this study, we have used the cell-permeable FP-N_3 probe conjugated to biotin via CuAAC-click chemistry and TMT-based isobaric labeling to provide the most in-depth study of *Plasmodium* SHs and quantify changes in their activity throughout the erythrocytic cycle in intact parasite.

In our study, we profiled the life-stage specific activity of 25 *Pf*SHs and 8 *Hs*SHs. Among the *Pf*SHs, we identified 22 out of 43 predicted α/βHs (Table 1), and 3 out of 4 predicted PLPs. SHs that are exported into the RBC/PV lumen were expected to be lost after saponin permeabilization. However, the identification of *Pf*EH1 and *Pf*XL2, which contain the *Plasmodium* PEXEL export element motifs (Hiller et al., 2004; Marti et al., 2004), suggests that some exported hydrolases are still detectable using our methods, especially if they are associated with the RBC membrane. Indeed, *Pf*EH1 has been shown to localize at the periphery of iRBCs and is associated with spectrin (Spillman et al., 2016). The remaining *Pf*SHs may not be expressed or might be below the detection limit. Alternatively, they may be present in an inactive form or within organelles inaccessible to FP-N_3. Finally, FP ABPs have been shown to target only 80–90% of metabolic SHs in complex biological samples (Kidd et al., 2001), and some of the predicted PfSHs might be pseudoenzymes (Zimmermann, 2013).

The majority of *Pf*SHs identified are unannotated, and for many, we have reported the first evidence of a serine nucleophile in their active site. All *Pf*SHs showed some change in activation during the erythrocytic life cycle. This suggests time-dependent activation that could be linked to the distinct metabolic roles and requirements of the different asexual life stages. The activation heat map presented here (Figure 3) is likely to be a useful tool in any further investigation into this large and relatively uncharacterized enzyme family. Of interest, some *Pf*SHs demonstrate a significant increase in activity levels between mature schizont and merozoite stages, for example Psta1 and RhoSH. Merozoite egress is a rapid and tightly regulated process, thus activity increases around this event are unlikely to be due to changes in protein expression or translation but rather specific activation. ABPP is well suited to detect these changes. RhoSH has recently been studied in our lab using conditional genetics. Conditional mutation of the catalytic triad His to Ala causes a defect in PVM formation immediately after invasion that results in a significant delay in intracellular development and a decrease in parasite replication (Dr S. Ridewood, personal communication). This is consistent with the subcellular localization of RhoSH in the rhoptries (apical secretory organelles that are important for invasion and PVM formation). The proposed function of RhoSH in PVM formation immediately after invasion is supported by the merozoite-specific activation shown here by ABPP.

In this study, four *Pf*SHs were selected for genetic validation in *P. falciparum*: abH112, abH114, Psta1 and Psta2. These candidates were prioritized based on their annotated essentiality or importance in high-throughput genetic screens (Zhang et al., 2018). Surprisingly, conditional mutation of their catalytic Ser showed no change in parasite replication. However, our conditional mutation results cannot rule out essential non-catalytic functions for these proteins. These *Pf*SHs could also have an essential role in lipid

metabolism *in vivo* that is not observable in cell culture, where parasites have an abundance of lipids and other nutrients in the media. Genes can be miss-annotated as essential in large scale genetic screens due to competition effects between co-cultured mutant lines. This has been reported before for other genes annotated as essential in the *piggyBac* screen such as the merozoite surface protein 1 (MSP1) or dipeptidyl aminopeptidase 3 (DPAP3) whose cKO only results in a 50% decrease in parasite replication (Das et al., 2015; Lehmann et al., 2018). By combining the wealth of data from large-scale genetic screening annotations with more targeted molecular biology approaches we can powerfully increase the quality of genome annotation.

One of the most interesting results from our ABPP study was the identification of 8 active *Hs*SHs in iRBCs. We demonstrate that inhibition of APEH, LYPLA1, FASN, PNPLA6 or AChE blocks parasite replication *in vitro*, with the APEH inhibitor AA74-1 and the LYPLA1 inhibitor ML348 being the most potent. From our ABPP results, LYPLA1, a predicted depalmitoylase, seems to be most active at ring and trophozoite stages. However, its inhibition only had an effect later in the cycle where a significant delay in schizogony and a block in egress was observed. *P. falciparum* employs palmitoylation machinery both in the parasite and RBC lumen, possibly both parasite and host derived (Kilian et al., 2020). LYPLA1 may be involved in this machinery. S-palmitoylated proteins have been shown to be abundant in the late trophozoite and schizont stages and essential for merozoite development (Jones et al., 2012; Kilian et al., 2020). Depalmitoylases in ring and trophozoite stage could be important in recycling palmitate ready for the later phases of asexual development. APEH is most active in merozoites, suggesting that it might be imported into the parasite and that it might play a role in invasion or early ring development. Interestingly, the APEH inhibitor displays sub-micromolar anti-parasitic activity and quickly kills parasites at ring stage. A recent article confirmed our hypothesis about the essentiality of APEH in asexual-blood stages and its import into the parasite (Elahi et al., 2019a). The endopeptidase activity of APEH, which cleaves N-acetylated amino acids from peptides, was suggested to play a role in the degradation of erythrocytic proteins in the parasite food vacuole (Elahi et al., 2019a).

These *Hs*SHs represent an exciting new area of research to develop anti-parasitic drugs. A number of other human proteins are thought to be co-opted by the parasite to perform functions such as haem biosynthesis (Bonday et al., 1997; Nagaraj et al., 2013; van Dooren et al., 2012). Host enzymes are attractive targets as they will not accumulate drug-resistance mutations. However, other mechanisms of resistance may arise, such as enzymatic inactivation of the drug or active efflux. Many human metabolic SHs have been pursued as promising drug targets for human diseases, such as AChE for Alzheimer's disease (Saxena and Dubey, 2019). This opens the possibility of repurposing *Hs*SH inhibitors as potential antimalarial drugs, although the toxicity of these inhibitors in humans has not been assessed. LYPLA1 inhibitor ML348 has no cytotoxicity effect in human embryonic kidney cells (HEK293T) up to 50μM (Adibekian et al., 2010a). Although AA74-1 has been shown to be highly selective for APEH in mice (4h treatment at 0.2–1.6mgkg^{-1}, intraperitoneal) mammalian toxicology studies have not been done (Adibekian et al., 2011). Finally, if the co-opted function of the host enzyme is essential for all malaria-causing *Plasmodium* species, the antimalarial drug should be equally effective as a treatment for all, as opposed to a drug that target a conserved parasite enzyme that might show different potencies among *Plasmodium* homologues. The targeting of human enzymes to fight infectious diseases has shown promise in virology. For example, human N-myristoyltransferases are co-opted for viron assembly, and their inhibition blocks virus replication and infectivity (Mousnier et al., 2018). This has been demonstrated in highly diverse viruses like Rhinovirus, responsible for the common cold, whose high replication and mutational rate have hampered any efforts to develop vaccines (Mousnier et al., 2018; Thibaut et al., 2012). Overall, targeting the host enzyme is a promising new avenue in the development of treatments against infective agents that rapidly acquire resistance to pathogen-directed drugs.

The study of human and *Plasmodium* metabolic SHs is a highly interesting field. Many *Pf*SHs are predicted to be lysophospholipases or lipases (Table 1), and therefore may play important roles in lipid and phospholipid scavenging, processing or synthesis. The phospholipid content of RBCs increases almost five-fold during intra-erythrocytic parasite growth, and the source of PVM lipids is still a topic of debate (Sherling and van Ooij, 2016; Tran et al., 2016). Lipids also play significant roles in signaling and immunogenic functions (Jiang et al., 2012). Mature erythrocytes do not perform *de novo* fatty acid synthesis because they lack acetyl-CoA carboxylase which provides the type 1 human fatty acid synthase (FASN) with malonyl-CoA (Pittman and Martin, 1966). The *Plasmodium*type II FASN pathway has been shown to be non-essential to asexual-blood stage replication (Vaughan et al., 2009). Therefore, the parasite may co-opt human

proteins to create a full fatty acid synthesis network in iRBCs. ABPP, metabolic chemical proteomics, lipidomics and metabolomics all have important roles to play in deciphering these complex biological processes and the interplay between host and parasite enzymes.

Genome annotation is of great importance in *Plasmodium* where only two thirds of the genes have any predicted function, few of which have been experimentally validated (Böhme et al., 2019). This study illustrates the benefits of ABPP-chemical proteomics to functionally annotate the genome based on a common catalytic mechanism. The distinction between active and non-active enzyme forms helps to align enzyme activity with life-stage specific functions, for instance the highly regulated events around merozoite egress. SH and protease ABPs were among the first to be described and certainly the most widely used (Greenbaum et al., 2000; Liu et al., 1999). Probes have also been developed towards glycosidases (Vocadlo and Bertozzi, 2004; Wu et al., 2019), phosphatases (Kalesh et al., 2010) and PLP-dependent enzymes (Hoegl et al., 2018). After the success of this in-depth ABPP study of SHs, we propose that ABPP should play a central role in the functional annotation of the *Plasmodium* genome.

Limitations of the study

A limitation of the LCMS-MS method is that lowly abundant proteins may be masked by larger peaks from highly abundant proteins, therefore causing us to miss some predicted *Pf*SHs. Ratio compression is also a problem when using isobaric labeling but we have attempted to minimize this by using fractionation to reduce sample complexity. We have used the soluble fraction of parasite lysates and despite the use of Triton-X100 there may be SHs in the insoluble fraction that have been missed. The reaction of a predicted SH with the FP probe does not necessarily mean that the catalytic activity of the SH is related or important to its main cellar role. This may be why the predicted essential *Pf*SHs we interrogated did not show any essentiality in our hands. There may also be limitations to our experimental model showing different essentialities than would emerge under less nutrient-rich conditions or *in vivo*.

When considering the activity changes of the *Hs*SHs, there is a limitation inherent in sample collection as there are many more human proteins present in ring stage- than in schizont stage-infected erythrocytes. Finally, although the *Hs*SH inhibitors that we have used all have demonstrated specificity in mammalian systems they could still have *P. falciparum* off-targets.

STAR★METHODS

Detailed methods are provided in the online version of this paper and include the following:

- KEY RESOURCES TABLE
- RESOURCE AVAILABILITY
  - Lead contact
  - Materials availability
  - Data and code availability
- EXPERIMENTAL MODEL AND SUBJECT DETAILS
- METHOD DETAILS
  - Parasite synchronization and preparation of parasite pellets
  - Labeling of SHs with FP-TMR
  - Parasite labeling for chemical proteomics
  - Affinity purification of labeled proteins and sample preparation for LCMS/MS
  - LCMS/MS data acquisition
  - LCMS/MS data analysis
  - Generation of conditional mutant lines
  - Western blot analysis
  - Parasite replication assay
  - Phenotypic characterization of *Hs*SHs inhibitor treatment by flow cytometry
- QUANTIFICATION AND STATISTICAL ANALYSIS

SUPPLEMENTAL INFORMATION

Supplemental information can be found online at https://doi.org/10.1016/j.isci.2022.104996.

ACKNOWLEDGMENTS

We would like to thank Prof M. Blackman and his group for their support, assistance and suggestions about this work, and for providing the B11 parasite cell lines. In particular, we would like to thank F. Hackett and M. Grainger for assistance in parasite culture and Prof E. Tate and his group for their advice and support on proteomic methods. We would also like to thank Dr. M. Treeck for the pT2A_FIKK10-cKO plasmid and Prof Y.T. Chang for the 132A RNA dye. This work was supported by a Marie Curie Career Integration Grant (PCIG14-GA-2013-631806) and by a Welcome Trust and Royal Society Sir Henry Dale Fellowship 099950. This work was also supported by the Francis Crick Institutewhich receives its core funding from Cancer Research UK (FC001043), the UK Medical Research Council (FC001043), and the Wellcome Trust (FC001043). For the purpose of open access, the authors have applied a CC BY public copyright license to any author accepted manuscript version arising from this submission.

AUTHOR CONTRIBUTIONS

Conceptualization, D.D. and E.D.; methodology, D.D. and S.H.; statistical analysis, D.D.; investigation, D.D.; writing, D.D.; editing, D.D. and E.D.; supervision, E.D. All authors read and approved the final manuscript.

DECLARATION OF INTERESTS

The authors declare no competing interests.

Received: February 15, 2022
Revised: March 14, 2022
Accepted: August 18, 2022
Published: September 16, 2022

REFERENCES

Adibekian, A., Martin, B.R., Chang, J.W., Hsu, K.-L., Tsuboi, K., Bachovchin, D.A., Speers, A.E., Brown, S.J., Spicer, T., et al. (2010a). Characterization of a selective, reversible inhibitor of lysophospholipase 1 (LYPLA1). In Probe Reports from the NIH Molecular Libraries Program (National Center for Biotechnology Information (US)).

Adibekian, A., Martin, B.R., Chang, J.W., Hsu, K.-L., Tsuboi, K., Bachovchin, D.A., Speers, A.E., Brown, S.J., Spicer, T., Fernandez-Vega, V., Ferguson, J., Cravatt, B.F., Hodder, P., and Rosen, H. (2010b). Characterization of a Selective, Reversible Inhibitor of Lysophospholipase 2 (LYPLA2). In Probe Reports from the NIH Molecular Libraries Program (National Center for Biotechnology Information (US)).

Adibekian, A., Martin, B.R., Chang, J.W., Hsu, K.-L., Tsuboi, K., Bachovchin, D.A., Speers, A.E., Brown, S.J., Spicer, T., Fernandez-Vega, V., et al. (2012). Confirming target engagement for reversible inhibitors *in vivo* by kinetically tuned activity-based probes. J. Am. Chem. Soc. *134*, 10345–10348. https://doi.org/10.1021/ja303400u.

Adibekian, A., Martin, B.R., Wang, C., Hsu, K.-L., Bachovchin, D.A., Niessen, S., Hoover, H., and Cravatt, B.F. (2011). Click-generated triazole ureas as ultrapotent *in vivo*–active serine hydrolase inhibitors. Nat. Chem. Biol. *7*, 469–478. https://doi.org/10.1038/nchembio.579.

Asahi, H., Kanazawa, T., Hirayama, N., and Kajihara, Y. (2005). Investigating serum factors promoting erythrocytic growth of Plasmodium falciparum. Exp. Parasitol. *109*, 7–15. https://doi.org/10.1016/j.exppara.2004.10.002.

Aurrecoechea, C., Brestelli, J., Brunk, B.P., Dommer, J., Fischer, S., Gajria, B., Gao, X., Gingle, A., Grant, G., Harb, O.S., et al. (2009). PlasmoDB: a functional genomic database for malaria parasites. Nucleic Acids Res. *37*, D539–D543. https://doi.org/10.1093/nar/gkn814.

Bachovchin, D.A., and Cravatt, B.F. (2012). The pharmacological landscape and therapeutic potential of serine hydrolases. Nat. Rev. Drug Discov. *11*, 52–68. https://doi.org/10.1038/nrd3620.

Bachovchin, D.A., Ji, T., Li, W., Simon, G.M., Blankman, J.L., Adibekian, A., Hoover, H., Niessen, S., and Cravatt, B.F. (2010). Superfamily-wide portrait of serine hydrolase inhibition achieved by library-versus-library screening. Proc. Natl. Acad. Sci. USA *107*, 20941–20946. https://doi.org/10.1073/pnas.1011663107.

Bannister, L.H., and Mitchell, G.H. (1989). The fine structure of secretion by Plasmodium knowlesi merozoites during red cell invasion. J. Protozool. *36*, 362–367. https://doi.org/10.1111/j.1550-7408.1989.tb05527.x.

Bell, D., Ridewood, S., Patel, A.P., Lee, S.H., Chang, Y.-T., and Deu, E. (2021). High content and high throughput phenotypic assay for the hourly resolution of the malaria parasite erythrocytic cycle. Preprint at bioRxiv. https://doi.org/10.1101/2021.03.15.434938.

Birnbaum, J., Flemming, S., Reichard, N., Soares, A.B., Mesén-Ramírez, P., Jonscher, E., Bergmann, B., and Spielmann, T. (2017). Selection Linked Integration (SLI) for Endogenous Gene Tagging and Knock Sideways in Plasmodium Falciparum Parasites.

Blackman, M.J. (1995). Chapter 11: purification of Plasmodium falciparum merozoites for analysis of the processing of merozoite surface protein-1. In Methods in Cell Biology, Microbes as Tools for Cell Biology, D.G. Russell, ed. (Academic Press), pp. 213–220. https://doi.org/10.1016/S0091-679X(08)61853-1.

Blackman, M.J. (2008). Malarial proteases and host cell egress: an 'emerging' cascade. Cell Microbiol. *10*, 1925–1934. https://doi.org/10.1111/j.1462-5822.2008.01176.x.

Böhme, U., Otto, T.D., Sanders, M., Newbold, C.I., and Berriman, M. (2019). Progression of the canonical reference malaria parasite genome from 2002-2019. Wellcome Open Res. *4*, 58. https://doi.org/10.12688/wellcomeopenres.15194.2.

Bonday, Z.Q., Dhanasekaran, S., Rangarajan, P.N., and Padmanaban, G. (2000). Import of host δ-aminolevulinate dehydratase into the malarial parasite: identification of a new drug target. Nat. Med. *6*, 898–903. https://doi.org/10.1038/78659.

Bonday, Z.Q., Taketani, S., Gupta, P.D., and Padmanaban, G. (1997). Heme biosynthesis by the malarial parasite. Import of delta-aminolevulinate dehydrase from the host red cell. J. Biol. Chem. *272*, 21839–21846. https://doi.org/10.1074/jbc.272.35.21839.

Bonventre, J.V., Huang, Z., Taheri, M.R., O'Leary, E., Li, E., Moskowitz, M.A., and Sapirstein, A. (1997). Reduced fertility and postischaemic brain injury in mice deficient in cytosolic phospholipase A2. Nature *390*, 622–625. https://doi.org/10.1038/37635.

CellPress
OPEN ACCESS

Bozdech, Z., Llinás, M., Pulliam, B.L., Wong, E.D., Zhu, J., and DeRisi, J.L. (2003). The transcriptome of the intraerythrocytic developmental cycle of Plasmodium falciparum. PLoS Biol. *1*, e5. https://doi.org/10.1371/journal.pbio.0000005.

Buchebner, M., Pfeifer, T., Rathke, N., Chandak, P.G., Lass, A., Schreiber, R., Kratzer, A., Zimmermann, R., Sattler, W., Koefeler, H., et al. (2010). Cholesteryl ester hydrolase activity is abolished in HSL macrophages but unchanged in macrophages lacking KIAA1363. J. Lipid Res. *51*, 2896–2908. https://doi.org/10.1194/jlr.M004259.

Bunkoczi, G., Misquitta, S., Wu, X., Lee, W.H., Rojkova, A., Kochan, G., Kavanagh, K.L., Oppermann, U., and Smith, S. (2009). Structural basis for different specificities of acyltransferases associated with the human cytosolic and mitochondrial fatty acid synthases. Chem. Biol. *16*, 667–675. https://doi.org/10.1016/j.chembiol.2009.04.011.

Burda, P.-C., Roelli, M.A., Schaffner, M., Khan, S.M., Janse, C.J., and Heussler, V.T. (2015). A Plasmodium phospholipase is involved in disruption of the liver stage parasitophorous vacuole membrane. PLoS Pathog. *11*, e1004760. https://doi.org/10.1371/journal.ppat.1004760.

Burrows, J.N., Duparc, S., Gutteridge, W.E., Hooft van Huijsduijnen, R., Kaszubska, W., Macintyre, F., Mazzuri, S., Möhrle, J.J., and Wells, T.N.C. (2017). New developments in anti-malarial target candidate and product profiles. Malar. J. *16*, 26. https://doi.org/10.1186/s12936-016-1675-x.

Burrows, J.N., van Huijsduijnen, R.H., Möhrle, J.J., Oeuvray, C., and Wells, T.N.C. (2013). Designing the next generation of medicines for malaria control and eradication. Malar. J. *12*, 187. https://doi.org/10.1186/1475-2875-12-187.

Carlton, J.M., Angiuoli, S.V., Suh, B.B., Kooij, T.W., Pertea, M., Silva, J.C., Ermolaeva, M.D., Allen, J.E., Selengut, J.D., Koo, H.L., et al. (2002). Genome sequence and comparative analysis of the model rodent malaria parasite Plasmodium yoelii yoelii. Nature *419*, 512–519. https://doi.org/10.1038/nature01099.

Cervantes, S., Prudhomme, J., Carter, D., Gopi, K.G., Li, Q., Chang, Y.-T., and Le Roch, K.G. (2009). High-content live cell imaging with RNA probes: advancements in high-throughput antimalarial drug discovery. BMC Cell Biol. *10*, 45. https://doi.org/10.1186/1471-2121-10-45.

Chang, J.W., Nomura, D.K., and Cravatt, B.F. (2011). A potent and selective inhibitor of KIAA1363/AADACL1 that impairs prostate cancer pathogenesis. Chem. Biol. *18*, 476–484. https://doi.org/10.1016/j.chembiol.2011.02.008.

Chang, J.W., Zuhl, A.M., Speers, A.E., Niessen, S., Brown, S.J., Mulvihill, M.M., Fan, Y.C., Spicer, T.P., Southern, M., Scampavia, L., et al. (2015). A selective inhibitor of platelet-activating factor acetylhydrolases 1b2 and 1b3 that impairs cancer cell survival. ACS Chem. Biol. *10*, 925–932. https://doi.org/10.1021/cb500893q.

Chang, S.I., and Hammes, G.G. (1990). Structure and mechanism of action of a multifunctional enzyme: fatty acid synthase. Acc. Chem. Res. *23*, 363–369. https://doi.org/10.1021/ar00179a003.

Chiang, K.P., Niessen, S., Saghatelian, A., and Cravatt, B.F. (2006). An enzyme that regulates ether lipid signaling pathways in cancer annotated by multidimensional profiling. Chem. Biol. *13*, 1041–1050. https://doi.org/10.1016/j.chembiol.2006.08.008.

Collins, C.R., Das, S., Wong, E.H., Andenmatten, N., Stallmach, R., Hackett, F., Herman, J.-P., Müller, S., Meissner, M., and Blackman, M.J. (2013a). Robust inducible Cre recombinase activity in the human malaria parasite Plasmodium falciparum enables efficient gene deletion within a single asexual erythrocytic growth cycle. Mol. Microbiol. *88*, 687–701. https://doi.org/10.1111/mmi.12206.

Collins, C.R., Hackett, F., Strath, M., Penzo, M., Withers-Martinez, C., Baker, D.A., and Blackman, M.J. (2013b). Malaria parasite cGMP-dependent protein kinase regulates blood stage merozoite secretory organelle discharge and egress. PLoS Pathog. *9*, e1003344. https://doi.org/10.1371/journal.ppat.1003344.

Cox, J., Neuhauser, N., Michalski, A., Scheltema, R.A., Olsen, J.V., and Mann, M. (2011). Andromeda: a peptide search engine integrated into the MaxQuant environment. J. Proteome Res. *10*, 1794–1805. https://doi.org/10.1021/pr101065j.

Cravatt, B.F., Wright, A.T., and Kozarich, J.W. (2008). Activity-based protein profiling: from enzyme chemistry to proteomic chemistry. Annu. Rev. Biochem. *77*, 383–414. https://doi.org/10.1146/annurev.biochem.75.101304.124125.

Das, S., Hertrich, N., Perrin, A.J., Withers-Martinez, C., Collins, C.R., Jones, M.L., Watermeyer, J.M., Fobes, E.T., Martin, S.R., Saibil, H.R., et al. (2015). Processing of Plasmodium falciparum merozoite surface protein MSP1 activates a spectrin-binding function enabling parasite egress from RBCs. Cell Host Microbe *18*, 433–444. https://doi.org/10.1016/j.chom.2015.09.007.

Davis, C.S., and Richardson, R.J. (1980). Organophosphorus compounds. In Experimental and Clinical Neurotoxicology, P.S. Spencer and H.H. Schaumburg, eds. (Philadelphia: Williams & Wilkins), pp. 527–544.

Dawson, R.M. (1994). Review of oximes available for treatment of nerve agent poisoning. J. Appl. Toxicol. *14*, 317–331. https://doi.org/10.1002/jat.2550140502.

Déchamps, S., Maynadier, M., Wein, S., Gannoun-Zaki, L., Maréchal, E., and Vial, H.J. (2010a). Rodent and nonrodent malaria parasites differ in their phospholipid metabolic pathways. J. Lipid Res. *51*, 81–96. https://doi.org/10.1194/jlr.M900166-JLR200.

Déchamps, S., Shastri, S., Wengelnik, K., and Vial, H.J. (2010b). Glycerophospholipid acquisition in Plasmodium - a puzzling assembly of biosynthetic pathways. Int. J. Parasitol. *40*, 1347–1365. https://doi.org/10.1016/j.ijpara.2010.05.008.

Deu, E. (2017). Proteases as antimalarial targets: strategies for genetic, chemical, and therapeutic validation. FEBS J. *284*, 2604–2628. https://doi.org/10.1111/febs.14130.

Deutsch, E.W., Bandeira, N., Sharma, V., Perez-Riverol, Y., Carver, J.J., Kundu, D.J., García-Seisdedos, D., Jarnuczak, A.F., Hewapathirana, S., Pullman, B.S., et al. (2020). The ProteomeXchange consortium in 2020: enabling 'big data' approaches in proteomics. Nucleic Acids Res. *48*, D1145–D1152. https://doi.org/10.1111/febs.14130.

Duncan, J.A., and Gilman, A.G. (1998). A cytoplasmic acyl-protein thioesterase that removes palmitate from G protein α subunits and p21RAS. J. Biol. Chem. *273*, 15830–15837. https://doi.org/10.1074/jbc.273.25.15830.

Elahi, R., Dapper, C., and Klemba, M. (2019a). Internalization of erythrocyte acylpeptide hydrolase is required for asexual replication of Plasmodium falciparum. mSphere *4*, e00077-19. https://doi.org/10.1128/mSphere.00077-19.

Elahi, R., Ray, W.K., Dapper, C., Dalal, S., Helm, R.F., and Klemba, M. (2019b). Functional annotation of serine hydrolases in the asexual erythrocytic stage of Plasmodium falciparum. Sci. Rep. *9*, 17532–17611. https://doi.org/10.1038/s41598-019-54009-0.

Elliott, D.A., McIntosh, M.T., Hosgood, H.D., Chen, S., Zhang, G., Baevova, P., and Joiner, K.A. (2008). Four distinct pathways of hemoglobin uptake in the malaria parasite Plasmodium falciparum. Proc. Natl. Acad. Sci. USA *105*, 2463–2468. https://doi.org/10.1073/pnas.0711067105.

Flammersfeld, A., Panyot, A., Yamaryo-Botté, Y., Aurass, P., Przyborski, J.M., Flieger, A., Botté, C., and Pradel, G. (2020). A patatin-like phospholipase functions during gametocyte induction in the malaria parasite Plasmodium falciparum. Cell Microbiol. *22*, e13146. https://doi.org/10.1111/cmi.13146.

Florens, L., Washburn, M.P., Raine, J.D., Anthony, R.M., Grainger, M., Haynes, J.D., Moch, J.K., Muster, N., Sacci, J.B., Tabb, D.L., et al. (2002). A proteomic view of the Plasmodium falciparum life cycle. Nature *419*, 520–526. https://doi.org/10.1038/nature01107.

Fujino, T., Watanabe, K., Beppu, M., Kikugawa, K., and Yasuda, H. (2000). Identification of oxidized protein hydrolase of human erythrocytes as acylpeptide hydrolase. Biochim. Biophys. Acta *1478*, 102–112.

Gómez-Díaz, E., Yerbanga, R.S., Lefèvre, T., Cohuet, A., Rowley, M.J., Ouedraogo, J.B., and Corces, V.G. (2017). Epigenetic regulation of Plasmodium falciparum clonally variant gene expression during development in Anopheles gambiae. Sci. Rep. *7*, 40655. https://doi.org/10.1038/srep40655.

Greenbaum, D., Medzihradszky, K.F., Burlingame, A., and Bogyo, M. (2000). Epoxide electrophiles as activity-dependent cysteine protease profiling and discovery tools. Chem. Biol. *7*, 569–581. https://doi.org/10.1016/S1074-5521(00)00014-4.

Groat-Carmona, A.M., Kain, H., Brownell, J., Douglass, A.N., Aly, A.S.I., and Kappe, S.H.I. (2015). A Plasmodium α/β-hydrolase modulates the development of invasive stages. Cell Microbiol. *17*, 1848–1867. https://doi.org/10.1111/cmi.12477.

Hattori, M., and Arai, H. (2015). Intracellular PAF-acetylhydrolase type I. The enzymes. Enzymes *38*,

23–36. https://doi.org/10.1016/bs.enz.2015.09.007.

Herkert, N.M., Thiermann, H., and Worek, F. (2011). *In vitro* kinetic interactions of pyridostigmine, physostigmine and soman with erythrocyte and muscle acetylcholinesterase from different species. Toxicol. Lett. *206*, 41–46. https://doi.org/10.1016/j.toxlet.2011.03.004.

Heuer, T.S., Ventura, R., Mordec, K., Lai, J., Fridlib, M., Buckley, D., and Kemble, G. (2016). FASN inhibition and taxane treatment combine to enhance anti-tumor efficacy in diverse xenograft tumor models through disruption of tubulin palmitoylation and microtubule organization and FASN inhibition-mediated effects on oncogenic signaling and gene expression. EBioMedicine *16*, 51–62. https://doi.org/10.1016/j.ebiom.2016.12.012.

Hiller, N.L., Bhattacharjee, S., van Ooij, C., Liolios, K., Harrison, T., Lopez-Estraño, C., and Haldar, K. (2004). A host-targeting signal in virulence proteins reveals a secretome in malarial infection. Science *306*, 1934–1937. https://doi.org/10.1126/science.1102737.

Hoegl, A., Nodwell, M.B., Kirsch, V.C., Bach, N.C., Pfanzelt, M., Stahl, M., Schneider, S., and Sieber, S.A. (2018). Mining the cellular inventory of pyridoxal phosphate-dependent enzymes with functionalized cofactor mimics. Nat. Chem. *10*, 1234–1245. https://doi.org/10.1038/s41557-018-0144-2.

Holmquist, M. (2000). Alpha/Beta-hydrolase fold enzymes: structures, functions and mechanisms. Curr. Protein Pept. Sci. *1*, 209–235.

Huesca, M., Al-Qawasmeh, R., Young, A.H., and Lee, Y. (2005). In Aryl Imidazoles and their Use as Anti-cancer Agents, WO2005047266A1.

Istvan, E.S., Mallari, J.P., Corey, V.C., Dharia, N.V., Marshall, G.R., Winzeler, E.A., and Goldberg, D.E. (2017). Esterase mutation is a mechanism of resistance to antimalarial compounds. Nat. Commun. *8*, 14240. https://doi.org/10.1038/ncomms14240.

Jessani, N., Niessen, S., Wei, B.Q., Nicolau, M., Humphrey, M., Ji, Y., Han, W., Noh, D.-Y., Yates, J.R., Jeffrey, S.S., and Cravatt, B.F. (2005). A streamlined platform for high-content functional proteomics of primary human specimens. Nat. Methods *2*, 691–697. https://doi.org/10.1038/nmeth778.

Jiang, H., Anderson, G.D., and McGiff, J.C. (2012). The red blood cell participates in regulation of the circulation by producing and releasing epoxyeicosatrienoic acids. Prostag. Other Lipid Mediat. *98*, 91–93. https://doi.org/10.1016/j.prostaglandins.2011.11.008.

Johnson, M.K. (1969). A phosphorylation site in brain and the delayed neurotoxic effect of some organophosphorus compounds. Biochem. J. *111*, 487–495. https://doi.org/10.1042/bj1110487.

Johnson, M.K. (1975). The delayed neuropathy caused by some organophosphorus esters: mechanism and challenge. CRC Crit. Rev. Toxicol. *3*, 289–316. https://doi.org/10.3109/10408447509079861.

Jones, M.L., Collins, M.O., Goulding, D., Choudhary, J.S., and Rayner, J.C. (2012). Analysis of protein palmitoylation reveals a pervasive role in Plasmodium development and pathogenesis. Cell Host Microbe *12*, 246–258. https://doi.org/10.1016/j.chom.2012.06.005.

Jullien, N., Sampieri, F., Enjalbert, A., and Herman, J.-P. (2003). Regulation of Cre recombinase by ligand-induced complementation of inactive fragments. Nucleic Acids Res. *31*, e131.

Kalesh, K.A., Tan, L.P., Lu, K., Gao, L., Wang, J., and Yao, S.Q. (2010). Peptide-based activity-based probes (ABPs) for target-specific profiling of protein tyrosine phosphatases (PTPs). Chem. Commun. *46*, 589–591. https://doi.org/10.1039/B919744C.

Kidd, D., Liu, Y., and Cravatt, B.F. (2001). Profiling serine hydrolase activities in complex proteomes. Biochemistry *40*, 4005–4015. https://doi.org/10.1021/bi002579j.

Kienesberger, P.C., Oberer, M., Lass, A., and Zechner, R. (2009). Mammalian patatin domain containing proteins: a family with diverse lipolytic activities involved in multiple biological functions. J. Lipid Res. *50*, S63–S68. https://doi.org/10.1194/jlr.R800082-JLR200.

Kilian, N., Zhang, Y., LaMonica, L., Hooker, G., Toomre, D., Mamoun, C.B., and Ernst, A.M. (2020). Palmitoylated proteins in Plasmodium falciparum-infected erythrocytes: investigation with click chemistry and metabolic labeling. Bioessays *42*, 1900145. https://doi.org/10.1002/bies.201900145.

Kim, J.H., Lee, S.-R., Li, L.-H., Park, H.-J., Park, J.-H., Lee, K.Y., Kim, M.-K., Shin, B.A., and Choi, S.-Y. (2011). High cleavage efficiency of a 2A peptide derived from porcine teschovirus-1 in human cell lines, zebrafish and mice. PLoS One *6*, e18556. https://doi.org/10.1371/journal.pone.0018556.

Kohnz, R.A., Mulvihill, M.M., Chang, J.W., Hsu, K.-L., Sorrentino, A., Cravatt, B.F., Bandyopadhyay, S., Goga, A., and Nomura, D.K. (2015). Activity-based protein profiling of oncogene-driven changes in metabolism reveals broad dysregulation of PAFAH1B2 and 1B3 in cancer. ACS Chem. Biol. *10*, 1624–1630. https://doi.org/10.1021/acschembio.5b00053.

Koncarevic, S., Rohrbach, P., Deponte, M., Krohne, G., Prieto, J.H., Yates, J., Rahlfs, S., and Becker, K. (2009). The malarial parasite Plasmodium falciparum imports the human protein peroxiredoxin 2 for peroxide detoxification. Proc. Natl. Acad. Sci. USA *106*, 13323–13328. https://doi.org/10.1073/pnas.0905387106.

Koster, R. (1946). Synergisms and antagonisms between physostigmine and di-isopropyl fluorophosphate in cats. J. Pharmacol. Exp. Ther. *88*, 39–46.

Kramer, K.J., Kan, S.C., and Siddiqui, W.A. (1982). Concentration of Plasmodium falciparum-infected erythrocytes by density gradient centrifugation in Percoll. J. Parasitol. *68*, 336–337.

Kumar, A., Kollath-Leiß, K., and Kempken, F. (2013). Characterization of bud emergence 46 (BEM46) protein: sequence, structural, phylogenetic and subcellular localization analyses. Biochem. Biophys. Res. Commun. *438*, 526–532. https://doi.org/10.1016/j.bbrc.2013.07.103.

Lambros, C., and Vanderberg, J.P. (1979). Synchronization of Plasmodium falciparum erythrocytic stages in culture. J. Parasitol. *65*, 418–420.

Lane, R.M., Potkin, S.G., and Enz, A. (2006). Targeting acetylcholinesterase and butyrylcholinesterase in dementia. Int. J. Neuropsychopharmacol. *9*, 101–124. https://doi.org/10.1017/S1461145705005833.

Lasonder, E., Green, J.L., Grainger, M., Langsley, G., and Holder, A.A. (2015). Extensive differential protein phosphorylation as intraerythrocytic Plasmodium falciparum schizonts develop into extracellular invasive merozoites. Proteomics *15*, 2716–2729. https://doi.org/10.1002/pmic.201400508.

Lehmann, C., Tan, M.S.Y., de Vries, L.E., Russo, I., Sanchez, M.I., Goldberg, D.E., and Deu, E. (2018). Plasmodium falciparum dipeptidyl aminopeptidase 3 activity is important for efficient erythrocyte invasion by the malaria parasite. PLoS Pathog. *14*, e1007031. https://doi.org/10.1371/journal.ppat.1007031.

Liu, Y., Patricelli, M.P., and Cravatt, B.F. (1999). Activity-based protein profiling: the serine hydrolases. Proc. Natl. Acad. Sci. USA *96*, 14694–14699. https://doi.org/10.1073/pnas.96.26.14694.

Long, J.Z., and Cravatt, B.F. (2011). The metabolic serine hydrolases and their functions in mammalian physiology and disease. Chem. Rev. *111*, 6022–6063. https://doi.org/10.1021/cr200075y.

López-Barragán, M.J., Lemieux, J., Quiñones, M., Williamson, K.C., Molina-Cruz, A., Cui, K., Barillas-Mury, C., Zhao, K., and Su, X.z. (2011). Directional gene expression and antisense transcripts in sexual and asexual stages of Plasmodium falciparum. BMC Genom. *12*, 587. https://doi.org/10.1186/1471-2164-12-587.

Mailu, B.M., Li, L., Arthur, J., Nelson, T.M., Ramasamy, G., Fritz-Wolf, K., Becker, K., and Gardner, M.J. (2015). Plasmodium apicoplast Gln-tRNAGln biosynthesis utilizes a unique GatAB amidotransferase essential for erythrocytic stage parasites. J. Biol. Chem. *290*, 29629–29641. https://doi.org/10.1074/jbc.M115.655100.

Marks, P.A., Gellhorn, A., and Kidson, C. (1960). Lipid synthesis in human leukocytes, platelets, and erythrocytes. J. Biol. Chem. *235*, 2579–2583.

Marti, M., Good, R.T., Rug, M., Knuepfer, E., and Cowman, A.F. (2004). Targeting malaria virulence and remodeling proteins to the host erythrocyte. Science *306*, 1930–1933. https://doi.org/10.1126/science.1102452.

Matera, A.G., and Wang, Z. (2014). A day in the life of the spliceosome. Nat. Rev. Mol. Cell Biol. *15*, 108–121. https://doi.org/10.1038/nrm3742.

Mercker, M., Kollath-Leiss, K., Allgaier, S., Weiland, N., and Kempken, F. (2009). The BEM46-like protein appears to be essential for hyphal development upon ascospore germination in Neurospora crassa and is targeted to the

endoplasmic reticulum. Curr. Genet. 55, 151–161. https://doi.org/10.1007/s00294-009-0232-3.

Mousnier, A., Bell, A.S., Swieboda, D.P., Morales-Sanfrutos, J., Pérez-Dorado, I., Brannigan, J.A., Newman, J., Ritzefeld, M., Hutton, J.A., Guedán, A., et al. (2018). Fragment-derived inhibitors of human N-myristoyltransferase block capsid assembly and replication of the common cold virus. Nat. Chem. 10, 599–606. https://doi.org/10.1038/s41557-018-0039-2.

Nagaraj, V.A., Sundaram, B., Varadarajan, N.M., Subramani, P.A., Kalappa, D.M., Ghosh, S.K., and Padmanaban, G. (2013). Malaria parasite-synthesized heme is essential in the mosquito and liver stages and complements host heme in the blood stages of infection. PLoS Pathog. 9, e1003522. https://doi.org/10.1371/journal.ppat.1003522.

Niphakis, M.J., and Cravatt, B.F. (2014). Enzyme inhibitor discovery by activity-based protein profiling. Annu. Rev. Biochem. 83, 341–377. https://doi.org/10.1146/annurev-biochem-060713-035708.

Oehring, S.C., Woodcroft, B.J., Moes, S., Wetzel, J., Dietz, O., Pulfer, A., Dekiwadia, C., Maeser, P., Flueck, C., Witmer, K., et al. (2012). Organellar proteomics reveals hundreds of novel nuclear proteins in the malaria parasite Plasmodium falciparum. Genome Biol. 13, R108. https://doi.org/10.1186/gb-2012-13-11-r108.

Okerberg, E.S., Wu, J., Zhang, B., Samii, B., Blackford, K., Winn, D.T., Shreder, K.R., Burbaum, J.J., and Patricelli, M.P. (2005). High-resolution functional proteomics by active-site peptide profiling. Proc. Natl. Acad. Sci. USA 102, 4996–5001. https://doi.org/10.1073/pnas.0501205102.

Ott, P. (1985). Membrane acetylcholinesterases: purification, molecular properties and interactions with amphiphilic environments. Biochim. Biophys. Acta 822, 375–392. https://doi.org/10.1016/0304-4157(85)90016-4.

Otto, T.D., Wilinski, D., Assefa, S., Keane, T.M., Sarry, L.R., Böhme, U., Lemieux, J., Barrell, B., Pain, A., Berriman, M., et al. (2010). New insights into the blood-stage transcriptome of Plasmodium falciparum using RNA-Seq. Mol. Microbiol. 76, 12–24. https://doi.org/10.1111/j.1365-2958.2009.07026.x.

Patricelli, M.P., Giang, D.K., Stamp, L.M., and Burbaum, J.J. (2001). Direct visualization of serine hydrolase activities in complex proteomes using fluorescent active site-directed probes. Proteomics 1, 1067–1071. https://doi.org/10.1002/1615-9861(200109)1:9<1067::AID-PROT1067>3.0.CO;2-4.

Paul, A.S., Egan, E.S., and Duraisingh, M.T. (2015). Host-parasite interactions that guide red blood cell invasion by malaria parasites. Curr. Opin. Hematol. 22, 220–226. https://doi.org/10.1097/MOH.0000000000000135.

Pelle, K.G., Oh, K., Buchholz, K., Narasimhan, V., Joice, R., Milner, D.A., Brancucci, N.M., Ma, S., Voss, T.S., Ketman, K., et al. (2015). Transcriptional profiling defines dynamics of parasite tissue sequestration during malaria infection. Genome Med. 7, 19. https://doi.org/10.1186/s13073-015-0133-7.

Pemble, C.W., Johnson, L.C., Kridel, S.J., and Lowther, W.T. (2007). Crystal structure of the thioesterase domain of human fatty acid synthase inhibited by Orlistat. Nat. Struct. Mol. Biol. 14, 704–709. https://doi.org/10.1038/nsmb1265.

Perez-Riverol, Y., Bai, J., Bandla, C., García-Seisdedos, D., Hewapathirana, S., Kamatchinathan, S., Kundu, D.J., Prakash, A., Frericks-Zipper, A., Eisenacher, M., et al. (2022). The PRIDE database resources in 2022: a Hub for mass spectrometry-based proteomics evidences. Nucleic Acids Res. 50, D543–D552.

Perrin, A.J., Collins, C.R., Russell, M.R.G., Collinson, L.M., Baker, D.A., and Blackman, M.J. (2018). The actinomyosin motor drives malaria par- asite red blood cell invasion but not egress. mBio 9. e00905-18–7511. https://doi.org/10.1128/mBio.00905-18.

Pieperhoff, M.S., Pall, G.S., Jimenez-Ruis, E., Das, S., Wong, E.H., Heng, J., Mueller, S., Blackman, M.J., and Meissner, M. (2014). Conditional U1 gene silencing in toxoplasma gondii. Preprint at bioRxiv. https://doi.org/10.1101/008649.

Pittman, J.G., and Martin, D.B. (1966). Fatty acid biosynthesis in human erythrocytes: evidence in mature erythrocytes for an incomplete long chain fatty acid synthesizing system. J. Clin. Invest. 45, 165–172.

Quistad, G.B., Barlow, C., Winrow, C.J., Sparks, S.E., and Casida, J.E. (2003). Evidence that mouse brain neuropathy target esterase is a lysophospholipase. Proc. Natl. Acad. Sci. USA 100, 7983–7987. https://doi.org/10.1073/pnas.1232473100.

Richardson, R.J., Hein, N.D., Wijeyesakere, S.J., Fink, J.K., and Makhaeva, G.F. (2013). Neuropathy target esterase (NTE): overview and future. Chem. Biol. Interact. 203, 238–244. https://doi.org/10.1016/j.cbi.2012.10.024.

Riglar, D.T., Richard, D., Wilson, D.W., Boyle, M.J., Dekiwadia, C., Turnbull, L., Angrisano, F., Marapana, D.S., Rogers, K.L., and Whitchurch, C.B. (2011). Super-resolution dissection of coordinated events during malaria parasite invasion of the human erythrocyte. Cell Host Microbe 9, 9–20. https://doi.org/10.1016/j.chom.2010.12.003.

Saldanha, C. (2017). Human erythrocyte acetylcholinesterase in health and disease. Molecules 22, E1499. https://doi.org/10.3390/molecules22091499.

Saxena, M., and Dubey, R. (2019). Target enzyme in Alzheimer's disease: acetylcholinesterase inhibitors. Curr. Top. Med. Chem. 19, 264–275. https://doi.org/10.2174/1568026619666190128125912.

Schwach, F., Bushell, E., Gomes, A.R., Anar, B., Girling, G., Herd, C., Rayner, J.C., and Billker, O. (2015). PlasmoGEM, a database supporting a community resource for large-scale experimental genetics in malaria parasites. Nucleic Acids Res. 43, D1176–D1182. https://doi.org/10.1093/nar/gku1143.

Sekiya, M., Osuga, J.-I., Igarashi, M., Okazaki, H., and Ishibashi, S. (2011). The role of neutral cholesterol ester hydrolysis in macrophage foam cells. J. Atheroscler. Thromb. 18, 359–364.

Shao, B., Lu, M., Katz, S.C., Varley, A.W., Hardwick, J., Rogers, T.E., Ojogun, N., Rockey, D.C., Dematteo, R.P., and Munford, R.S. (2007). A host lipase detoxifies bacterial lipopolysaccharides in the liver and spleen. J. Biol. Chem. 282, 13726–13735. https://doi.org/10.1074/jbc.M609462200.

Sherling, E.S., and van Ooij, C. (2016). Host cell remodeling by pathogens: the exomembrane system in Plasmodium-infected erythrocytes. FEMS Microbiol. Rev. 40, 701–721. https://doi.org/10.1093/femsre/fuw016.

Silvestrini, F., Lasonder, E., Olivieri, A., Camarda, G., van Schaijk, B., Sanchez, M., Younis Younis, S., Sauerwein, R., and Alano, P. (2010). Protein export marks the early phase of gametocytogenesis of the human malaria parasite Plasmodium falciparum. Mol. Cell. Proteomics 9, 1437–1448. https://doi.org/10.1074/mcp.M900479-MCP200.

Singh, S., Plassmeyer, M., Gaur, D., and Miller, L.H. (2007). Mononeme: a new secretory organelle in Plasmodium falciparum merozoites identified by localization of rhomboid-1 protease. Proc. Natl. Acad. Sci. USA 104, 20043–20048. https://doi.org/10.1073/pnas.0709999104.

Soreq, H., and Seidman, S. (2001). Acetylcholinesterase — new roles for an old actor. Nat. Rev. Neurosci. 2, 294–302. https://doi.org/10.1038/35067589.

Spillman, N.J., Dalmia, V.K., and Goldberg, D.E. (2016). Exported epoxide hydrolases modulate erythrocyte vasoactive lipids during Plasmodium falciparum infection. mBio 7. e01538-16–16. https://doi.org/10.1128/mBio.01538-16.

Sugimoto, H., Hayashi, H., and Yamashita, S. (1996). Purification, cDNA cloning, and regulation of lysophospholipase from rat liver. J. Biol. Chem. 271, 7705–7711. https://doi.org/10.1074/jbc.271.13.7705.

Templeton, T.J. (2009). The varieties of gene amplification, diversification and hypervariability in the human malaria parasite, Plasmodium falciparum. Mol. Biochem. Parasitol. 166, 109–116. https://doi.org/10.1016/j.molbiopara.2009.04.003.

Thibaut, H.J., De Palma, A.M., and Neyts, J. (2012). Combating enterovirus replication: state-of-the-art on antiviral research. Biochem. Pharmacol. 83, 185–192. https://doi.org/10.1016/j.bcp.2011.08.016.

van Tienhoven, M., Atkins, J., Li, Y., and Glynn, P. (2002). Human neuropathy target esterase catalyzes hydrolysis of membrane lipids. J. Biol. Chem. 277, 20942–20948. https://doi.org/10.1074/jbc.M200330200.

Toenhake, C.G., Fraschka, S.A.-K., Vijayabaskar, M.S., Westhead, D.R., van Heeringen, S.J., and Bártfai, R. (2018). Chromatin accessibility-based characterization of the gene regulatory network underlying Plasmodium falciparum blood-stage development. Cell Host Microbe 23, 557–569.e9. https://doi.org/10.1016/j.chom.2018.03.007.

Tran, P.N., Brown, S.H.J., Rug, M., Ridgway, M.C., Mitchell, T.W., and Maier, A.G. (2016). Changes in lipid composition during sexual development of the malaria parasite Plasmodium falciparum. Malar. J. 15, 73. https://doi.org/10.1186/s12936-016-1130-z.

Tsunasawa, S., Narita, K., and Ogata, K. (1975). Purification and properties of acylamino acid-releasing enzyme from rat liver. J. Biochem. 77, 89–102.

Tyanova, S., Temu, T., and Cox, J. (2016a). The MaxQuant computational platform for mass spectrometry-based shotgun proteomics. Nat. Protoc. *11*, 2301–2319. https://doi.org/10.1038/nprot.2016.136.

Tyanova, S., Temu, T., Sinitcyn, P., Carlson, A., Hein, M.Y., Geiger, T., Mann, M., and Cox, J. (2016b). The Perseus computational platform for comprehensive analysis of (prote)omics data. Nat. Methods *13*, 731–740. https://doi.org/10.1038/nmeth.3901.

van Dooren, G.G., Kennedy, A.T., and McFadden, G.I. (2012). The use and abuse of heme in apicomplexan parasites. Antioxid. Redox Signal. *17*, 634–656. https://doi.org/10.1089/ars.2012.4539.

Vaughan, A.M., O'neill, M.T., Tarun, A.S., Camargo, N., Phuong, T.M., Aly, A.S.I., Cowman, A.F., and Kappe, S.H.I. (2009). Type II fatty acid synthesis is essential only for malaria parasite late liver stage development. Cell Microbiol. *11*, 506–520. https://doi.org/10.1111/j.1462-5822.2008.01270.x.

Ventura, R., Mordec, K., Waszczuk, J., Wang, Z., Lai, J., Fridlib, M., Buckley, D., Kemble, G., and Heuer, T.S. (2015). Inhibition of de novo Palmitate Synthesis by Fatty Acid Synthase Induces Apoptosis in Tumor Cells by Remodeling Cell Membranes, Inhibiting Signaling Pathways, and Reprogramming Gene Expression. EBioMedicine *2*, 808–824. https://doi.org/10.1016/j.ebiom.2015.06.020.

Veronesi, B., Padilla, S., Blackmon, K., and Pope, C. (1991). Murine susceptibility to organophosphorus-induced delayed neuropathy (OPIDN). Toxicol. Appl. Pharmacol. *107*, 311–324. https://doi.org/10.1016/0041-008x(91)90211-v.

Vocadlo, D.J., and Bertozzi, C.R. (2004). A strategy for functional proteomic analysis of glycosidase activity from cell lysates. Angew. Chem. Int. Ed. Engl. *43*, 5338–5342. https://doi.org/10.1002/anie.200454235.

WHO (2018). WHO|Global Report on Insecticide Resistance in Malaria Vectors: 2010–2016 (WHO). http://www.who.int/malaria/publications/atoz/9789241514057/en/.

WHO (2020). WHO | World malaria report 2020. https://www.who.int/publications-detail-redirect/9789240015791.

Willems, L.I., Overkleeft, H.S., and van Kasteren, S.I. (2014). Current developments in activity-based protein profiling. Bioconjug. Chem. *25*, 1181–1191. https://doi.org/10.1021/bc500208y.

Wilson, S.K., and Knoll, L.J. (2018). Patatin-like phospholipases in microbial infections with emerging roles in fatty acid metabolism and immune regulation by Apicomplexa. Mol. Microbiol. *107*, 34–46. https://doi.org/10.1111/mmi.13871.

Won, S.J., Davda, D., Labby, K.J., Hwang, S.Y., Pricer, R., Majmudar, J.D., Armacost, K.A., Rodriguez, L.A., Rodriguez, C.L., Chong, F.S., et al. (2016). Molecular mechanism for isoform-selective inhibition of acyl protein thioesterases 1 and 2 (APT1 and APT2). ACS Chem. Biol. *11*, 3374–3382. https://doi.org/10.1021/acschembio.6b00720.

Wu, L., Armstrong, Z., Schröder, S.P., de Boer, C., Artola, M., Aerts, J.M., Overkleeft, H.S., and Davies, G.J. (2019). An overview of activity-based probes for glycosidases. Curr. Opin. Chem. Biol. *53*, 25–36. https://doi.org/10.1016/j.cbpa.2019.05.030.

Yoo, E., Schulze, C.J., Stokes, B.H., Onguka, O., Yeo, T., Mok, S., Gnädig, N.F., Zhou, Y., Kurita, K., Foe, I.T., et al. (2020). The antimalarial natural product salinipostin A identifies essential α/β serine hydrolases involved in lipid metabolism in P. Falciparum parasites. Cell Chem. Biol. *27*, 143–157.e5. https://doi.org/10.1016/j.chembiol.2020.01.001.

Zanghì, G., Vembar, S.S., Baumgarten, S., Ding, S., Guizetti, J., Bryant, J.M., Mattei, D., Jensen, A.T.R., Rénia, L., Goh, Y.S., et al. (2018). A specific PfEMP1 is expressed in P. Falciparum sporozoites and plays a role in hepatocyte infection. Cell Rep. *22*, 2951–2963. https://doi.org/10.1016/j.celrep.2018.02.075.

Zhang, M., Wang, C., Otto, T.D., Oberstaller, J., Liao, X., Adapa, S.R., Udenze, K., Bronner, I.F., Casandra, D., Mayho, M., et al. (2018). Uncovering the essential genes of the human malaria parasite Plasmodium falciparum by saturation mutagenesis. Science *360*, eaap7847. https://doi.org/10.1126/science.aap7847.

Zimmermann, M. (2013). Neuronal AChE splice variants and their non-hydrolytic functions: redefining a target of AChE inhibitors? Br. J. Pharmacol. *170*, 953–967. https://doi.org/10.1111/bph.12359.

Zuccala, E.S., and Baum, J. (2011). Cytoskeletal and membrane remodelling during malaria parasite invasion of the human erythrocyte. Br. J. Haematol. *154*, 680–689. https://doi.org/10.1111/j.1365-2141.2011.08766.x.

STAR★METHODS

KEY RESOURCES TABLE

REAGENT or RESOURCE	SOURCE	IDENTIFIER
Antibodies		
Anti-HA-Biotin, High Affinity (3F10)	Roche	Cat#12158167001; RRID:AB_390915
Horseradish peroxidase conjugated Goat anti Rat IgG antibody	Bio-Rad	Cat#5201–2504
Anri-Lysophospholipase 1 antibody [EPR3667]	Abcam	Cat#ab91606; RRID:AB_10565192
Anti-APEH antibody	Atlas antibodies	Cat#HPA029700; RRID:AB_10599278
Anti-Biotin antibody produced in goat	Sigma	Cat#B3640; RRID:AB_258552
Bacterial and virus strains		
XL10-Gold Ultracompetent cells	Aligent	Cat#200315
Biological samples		
Anonymized human blood	United Kingdom National Health System Blood and Transplant Special Health Authority	N/A
Chemicals, peptides, and recombinant proteins		
TMT Mass Tagging reagents	Thermo Fisher Scientific	Cat#90308/90309 Lot#RH239931/239932
ActivX™ TAMRA-FP Serine Hydrolase Probe	Thermo Fisher Scientific	Cat#88318
ActivX™ Azido-FP Serine Hydrolase Probe	Thermo Fisher Scientific	Cat#88316
Compound 2 (C2)	Kind gift of Dr Simon Osbourne, LifeArc, SBC Open Innovation Campus, Stevenage	n/a
Biotin-alkyne	Jena Bioscience	Cat#CLK-1134
Tris(2-carboxyethyl)phosphine hydrochloride (TCEP)	Aldrich	Cat#C4706
tris[(1-benzyl-1H-1,2,3-triazol-4-yl)methyl]amine (TBTA)	Sigma	Cat#678937
Trypsin Gold	Promega	Cat#V5280
SalI restriction endonuclease	New England Bioscience	Cat#R0138
BglII restriction endonuclease	New England Bioscience	Cat#R0144
Phusion® High-Fidelity DNA Polymerase	New England Bioscience	Cat#M0530
Geneticin selective Antibiotic G418 Sulphate	Gibco	Cat#11811023
ML348	Sigma	Cat#SML1901
ML349	Sigma	Cat#SML1918
AA74-1	Sigma	Cat#CAY17507
P11	Cayman Chemical	Cat#CAY17507
Pyridostigmine bromide (PyBr)	Sigma	Cat#P9797
Tri-o-cresyl phosphate (TOCP)	Supelco	Cat#51885
JW480	Sigma	Cat#SML17507
TBV-3166	Sigma	Cat#SML1694
132A RNA dye	Kind gift of Prof YT Chang, National University of Singapore	n/a

(Continued on next page)

Continued		
REAGENT or RESOURCE	SOURCE	IDENTIFIER
Pierce NeutrAvidin Agarose	Thermo Fisher Scientific	Cat#29200
Pierce Control Agarose resin	Thermo Fisher Scientific	Cat#26150
RMPI-1640 based media supplemented with 0.292 g/L L-glutamine, 25 μg/ml gentamycin and 5% (w/v) Albumax II	Gibco	Cat#04191762A
Percoll	GE Healthcare Life Science	Cat#17-0891-01
Pierce BCA Protein Assay Kit	Thermo Fisher Scientific	Cat#23225
Empore Octadecyl C18 47mm Extraction Disks 2215	Supleco	Cat#66883-U
pH reversed-phase peptide fractionation kit	Pierce	Cat#84868
WR99210	Jacobus Pharmaceuticals	n/a
Infusion HD Cloning Kit	ClonTech	Cat#102518
DNeasy Blood and Tissue Kit	Qiagen	Cat#69504
QIAquick PCR purification kit	Qiagen	Cat#28104
SYBR Safe	Thermo Fisher Scientific	Cat#S33102
QIAprep Midi Kit	Qiagen	Cat#12943
P3 Primary cell 4-D Nucleofector X kit	Lonza	Cat#V4XP-3034
Deposited data		
Life cycle proteomics data	ProteomeXchange Consortium via PRIDE. http://www.ebi.ac.uk/pride	**Project accession** PRIDE: PXD035891
Experimental models: Cell lines		
P. falciparum 3D7	Professor Mike Blackman, The Francis Crick Instiutue	n/a
P. falciparum B11	Professor Mike Blackman, The Francis Crick Instiutue (Perrin et al., 2018)	n/a
P. falciparum Psta1-cMUT	This paper	n/a
P. falciparum Psta2-cMUT	This paper	n/a
P. falciparum abH112-cMUT	This paper	n/a
P. falciparum abH114-cMUT	This paper	n/a
Oligonucleotides		
Primers for *Pf* cMUT generation	See Table S2 for list of primers and sequences	
Recombinant DNA		
pT2A-Psta1-cMUT	This paper (adapted from pT2A_FIKK10_cKO gift of Dr M. Treeck, The Francis Crick Institute)	n/a
pT2A-Psta2-cMUT	This paper (adapted from pT2A_FIKK10_cKO gift of Dr M. Treeck, The Francis Crick Institute)	n/a
pT2A-ab112-cMUT	This paper (adapted from pT2A_FIKK10_cKO gift of Dr M. Treeck, The Francis Crick Institute)	n/a
pT2A-ab114-cMUT	This paper (adapted from pT2A_FIKK10_cKO gift of Dr M. Treeck, The Francis Crick Institute)	n/a
Psta1 synthetic DNA for the generation of pT2A-Psta1-cMUT	GENEWIZ	n/a

(*Continued on next page*)

Continued

REAGENT or RESOURCE	SOURCE	IDENTIFIER
Psta2 synthetic DNA for the generation of pT2A-Psta2-cMUT	GENEWIZ	n/a
abH112 synthetic DNA for the generation of pT2A-abH112-cMUT	GENEWIZ	n/a
abH114 synthetic DNA for the generation of pT2A-abH112-cMUT	GENEWIZ	n/a
Software and algorithms		
MaxQuant v1.6.0.1	Max-Planck-Institute of Biochemistry	https://maxquant.org
Perseus v1.5.6.0	Max-Planck-Institute of Biochemistry	https://maxquant.org
FACSDiva software v8.0.1	BD biosciences	https://www.bdbiosciences.com/en-ca/products/software/instrument-software/bd-facsdiva-software#Overview
FlowJo 2006-2015	FlowJo LLC	https://www.flowjo.com
Prism v7	GraphPad	https://www.graphpad.com/

RESOURCE AVAILABILITY

Lead contact

Further information and requests for resources and reagents should be directed to and will be fulfilled by the lead contact, Dara Davison d.davison@ucl.ac.uk.

Materials availability

Plasmids and cell lines generated in this study are available from the lead contact on request.

Data and code availability

The mass spectrometry proteomics data have been deposited to the ProteomeXchange Consortium (Deutsch et al., 2020) via the PRIDE (Perez-Riverol et al., 2022) partner repository, with the dataset identifier PRIDE: PXD035891, and are publicly available as of the date of publication. Raw flow cytometry data and original western blot and DNA/protein gel images reported in this article will be shared by the lead contact on request.

This article does not report original code.

Any additional information required to reanalyze the data reported in this article is available from the lead contact on request.

EXPERIMENTAL MODEL AND SUBJECT DETAILS

Anonymized human blood to culture malaria parasites was purchased from the United Kingdom National Health System Blood and Transplant Special Health Authority. No ethical approval is required for its use.

Pf WT 3D7 and *Pf* B11 (Perrin et al., 2018) were cultured at 37°C in 1–4% hematocrit of human erythrocytes under an atmosphere of 90% N_2, 5% CO_2, 5% O_2 (Sherling and van Ooij, 2016; Tran et al., 2016) and using RMPI-1640 (Gibco) based media supplemented with 0.292 g/L L-glutamine, 25 µg/mL gentamycin and 5% (w/v) Albumax II.

METHOD DETAILS

Parasite synchronization and preparation of parasite pellets

Synchronization of parasite cultures was achieved by isolating mature schizonts by density gradient centrifugation using a 63% Percoll (GE Healthcare Life Science) cushion (Kramer et al., 1982). Schizonts were then allowed to invade fresh erythrocytes for 2h in complete medium under shaking conditions. Remaining schizonts were removed by using a second Percoll purification step, and the remaining erythrocytes treated with 5% D-sorbitol (Lambros and Vanderberg, 1979) to leave a synchronous culture of ring-stage parasites. When required, parasites lines were cryopreserved in liquid N_2 by freezing erythrocytes of ring-stage

cultures at 1–5% parasitemia suspended in one volume of freezing solution (28% (v/v) glycerol, 3% (w/v) sorbitol, 65% (w/v) NaCl in dH_2O). To thaw cryopreserved parasite lines, cryovials were warmed at 37°C before the contents was washed twice with 5 mL of thawing solution (3.4% (w/v) NaCl in dH_2O). Parasitemia and parasite morphology were routinely visualized by light microscopy in Giemsa-stained (VWR International) thin blood smears.

Collection of parasite pellets at different stages was performed as follows. Schizonts were isolated via Percoll purification, the RBC and PV membrane permeabilized with 0.15% saponin (5 min at 37°C), and the pellets washed once with PBSa (137 mM NaCl, 2.7 mM KCl, 10 mM Na_3PO_4, pH 7.2) before freezing and liquid N2 and storage at −80°C. To collect mature segmented schizonts, schizonts were Percoll purified from a synchronous culture at the time of egress and treated for 3-4h in the presence of 1 μM C2 (A kind gift of Dr Simon Osbourne, LifeArc, SBC Open Innovation Campus, Stevenage, UK) (Collins et al., 2013b). Ring and trophozoite pellets were obtained after saponin permeabilization of erythrocytes from synchronous cultures. Pellets were washed thrice with PBSa to remove as much hemoglobin and RBC debris as possible, frozen in liquid N_2 and stored at −80°C. Merozoites were collected from an actively egressing culture of Percoll-purified schizonts (Blackman, 1995) at 37°C and under shaking conditions in RPMI-1640 media (Gibco) supplemented with 5% horse serum and 0.292 g/L L-glutamine. Unruptured schizonts were separated from merozoites by multiple rounds of centrifugation (1200 rpm for 3 min). The supernatant containing the merozoites was passed through a magnet (SuperMACS) to remove residual schizonts and hemozoin-associated erythrocyte debris. The merozoites were then pelleted by fast centrifugation (17,000 × g), frozen in liquid N_2 and stored at −80°C.

Lysates were prepared by diluting parasite pellets in one volume of 0.1% Triton X-100 (Promega) in PBSa and incubating the mixture on ice for 20 min. The soluble protein fraction was collected after centrifugation. Protein concentration in parasite lysates was measured using a NanoDrop 2000 spectrophotometer (Thermo Fisher Scientific) or a Pierce™ BCA Protein Assay Kit (Thermo Fisher Scientific) following the manufacturer's instructions.

Labeling of SHs with FP-TMR

Parasite lysates were diluted to 2 mg/mL in PBSa before labeling with 0.1 to 1 μM of FP-TMR (Thermo Fisher Scientific) for 30 to 60 min. Labeling reactions were quenched by addition of 4X Laemmli buffer (LB, 10% glycerol, 2% SDS, 67.5 mM Tris-HCl, 0.005% bromophenol blue, and 400 mM dithiothreitol (DTT) and heating samples at 95°C for 5 min. After SDS-PAGE of the samples, in-gel fluorescence was measured using a Bio-Rad PharosFX fluorescence scanner (552 nm excitation laser, 572 nm emission filter). In competition assays, parasite lysates were pretreated with *Hs*SH inhibitors (0.2 μM ML348 or 17 μM AA74-1) for 30 min before adding 1 μM or 50 nM FP-TMR for 10 min.

Parasite labeling for chemical proteomics

For all steps of proteomics protocols, 1.5 mL Eppendorf® LoBind microcentrifuge tubes (Sigma) were used to minimize protein losses. To label SHs in lysates, parasite pellets were resuspended in one volume of 0.1% Triton X-100 (Promega) in proteomic grade PBS (pH 7.4, Invitrogen, supplied as 10X and diluted to 1X in ddH_2O Pierce LC-MS grade) and incubated on ice for 20 min. The soluble fraction was recovered by centrifugation and protein concentration determined using a Pierce™ BCA Protein Assay Kit (23225, Thermo Fisher Scientific) following the manufacturer's instructions. Lysates were diluted to 4 mg/mL in PBS, and 600 μg of protein labeled for 1h with 1 μM of FP-Biotin or FP-N_3. The reaction was quenched by the addition of Triton X-100 and SDS to a final concentration of 2 and 0.2% in PBS, respectively.

For labeling SHs in intact parasites with FP-N_3, erythrocytes from ring and trophozoite stage cultures, and Percoll-purified early and mature schizonts, were diluted 10-fold in RPMI-1640 and incubated for 1h with 1 μM FP-N_3 at RT with gentle shaking. Note that ring, trophozoite and early schizont stages were roughly synchronized by increasing the invasion time from 2 to 5 h. For ring stage parasites, a time window of 0–20 hpi was achieved by mixing two ring populations that had been synchronized 10 h apart. This was not deemed necessary to obtain trophozoites (20–36 hpi) or early schizonts (36–44 hpi) as parasites diverge from synchronicity slightly throughout the life cycle. Highly mature schizonts were obtained following C2 treatment and labeled in the presence of C2. After probe treatment, parasites were washed twice with PBSa to remove excess probe and treated with 0.15% saponin in PBSa for 5 min at 37°C. The pellet was washed with PBSa, once for schizonts and thrice for rings and trophozoites, and snap frozen in liquid N_2.

Soluble protein lysate was then extracted as described above and diluted into 2% Triton X-100 and 0.2% SDS in PBS to a final concentration of 4 mg/mL before biotin conjugation via click chemistry. Note that labeling of SHs in merozoites was performed in lysates.

To conjugate biotin to FP-N_3, treated samples were diluted in one volume of PBS (final volume of 282 μL) and added to 18 μL of CuAAC click chemistry reagents, which were mixed by sequentially adding 3 μL of 10 mM biotin-alkyne (Jena Bioscience), 6 μL of 50 mM $CuSO_4$, 6 μL of 50 mM Tris (2-carboxyethyl)phosphine hydrochloride (TCEP, Aldrich), and 3 μL of 10 mM tris[(1-benzyl-1H-1,2,3-triazol-4-yl)methyl]amine (TBTA, Sigma). Final concentrations: 100 μM biotin-alkyne, 1 mM $CuSO_4$, 1 mM TCEP, 100 μM TBTA. After 1h incubation at RT with gentle mixing, the reaction was quenched by adding 3.3 μL of 5 mM EDTA. Excess probe and reagents were removed by MeOH/$ClCH_3$ protein precipitation (2 volumes MeOH (Thermo Fisher Scientific), 0.5 volumes $CHCL_3$ (Thermo Fisher Scientific) and 1 volume ddH_2O (Pierce LC-MS grade)), centrifugation at 17,000 × g for 2 min, wash with 1mL MeOH, and air drying in a fume hood. Protein was resolubilized by vortexing for 15 min at RT in 2% SDS (Sigma) and 10 mM DTT in PBS.

Affinity purification of labeled proteins and sample preparation for LCMS/MS

Protein samples were diluted to 1 mg/mL in 600 μL of 0.2% SDS and 1 mM DTT in PBS, and incubated for 2h with a mixture of 10 μL NeutrAvidin (Thermo Fisher Scientific) and 20 μL blank (Pierce) agarose bead slurries. The bead-mix was centrifuged (3,000 × g, 2 min) to collect the supernatant, and then washed sequentially with 3 × 1% SDS in PBS, 2 × 4 M urea in 50 mM ammonium bicarbonate (AMBIC, BioUltra 99.5% Sigma in dH_2O) and 2 × AMBIC.

Proteins were then treated to reduce and alkylate free thiols. Beads were treated with 10 mM TCEP in AMBIC for 1 h at 37°C, washed with AMBIC, and then treated with 10 mM N-ethylmaleimide (NEM, Sigma) in AMBIC for 30 min at RT before being washed again with AMBIC.

On-bead digestion was performed by adding 0.12 μg Trypsin Gold (Promega) diluted in 50 μL of AMBIC and overnight incubation at 37°C under shaking conditions. The beads were then washed with 70 μL AMBIC and 70 μL 1.5% trifluoroacetic acid (TFA, Thermo Fisher Scientific) in ddH_2O, and the flowthroughs combined. The peptide mixture was filtered and desalted on homemade 'stage tips' containing C18 membrane (Empore Octadecyl C18 47mm Extraction Disks 2215 Supleco 66883-U). Tips were washed by adding 150 μL MeOH and 150 μL ddH_2O and centrifuging for 2 min at 2000 × g. The peptide sample (140 μL) was loaded (2000 × g, 1 min) and the tips washed with 150 μL ddH_2O. Peptides were eluted with 60 μL 40% acetonitrile (ACN, 99.8% anhydrous, Sigma) in ddH_2O (2000 × g, 1 min). The peptide solution was then evaporated to dryness using a speed vacuum (Savant SpeedVac Integrated system ISS 100).

Peptide samples were dissolved in 25 μL of 50 mM triethylammonium bicarbonate buffer (TEAB, Sigma). 0.2 mg of TMT label reagents (Thermo Fisher Scientific TMTsixplex™ product number 90308, lot numberRH239931 or TMTtenplex™ product number 90309, lot numberRH239932) were dissolved in 65 μL anhydrous ACN. 20 μL of the appropriate TMT label was added to each replicate, and samples incubated for 1h at RT. To check labeling efficiency, 1 μL of TMT labeled sample was added to 49 μL of 5% and 0.1% TFA and analyzed by LC-MS/MS. All TMT-six/tenplex labeled samples were combined into one sample for each replicate and separated into 8 fractions using a high pH reversed-phase peptide fractionation kit (Pierce 84868). Fractionated samples were then evaporated to dryness by vacuum centrifugation (CentriVap Concentrator, Labconco).

LCMS/MS data acquisition

The peptide mixture was resuspended in 15 μL 0.1% TFA; 10 μL were used per injection unless otherwise stated. Peptides were chromatographically resolved on an UltiMate 3000 nanoRSLC HPLC (Thermo Fisher Scientific). A 2 × 0.3 mm Acclaim Pepmap C18 trap column (Thermo Fisher Scientific) was used at a flow rate of 15 μL/min before the trap being switched to elute at 0.25 μL/min through a 50 cm × 75 μm EasySpray C18 column. In a 90 min run the following gradients were used: 9–25% solution B over 37 min, 25–40% B over 18 min, a short gradient to 100% B, and finally, a 20 min equilibration in 9% B (A = 2% ACN, 0.1% formic acid; B = 80% ACN, 0.1% formic acid). HPLC eluent was introduced into an Orbitrap Fusion Lumos (Thermo Fisher Scientific). The Orbitrap was operated in "Data Dependent Acquisition" mode with a survey scan at a resolution of 120,000 from m/z 400–1400. This was followed by MS/MS using 38% high energy collision dissociation (HCD). Dynamic exclusion was used with a time window of 30s.

LCMS/MS data analysis

The raw data files were analyzed using MaxQuant version 1.6.0.1 (Tyanova et al., 2016a). Quantification was done at MS2 level using TMT-six/tenplex labels. All other MaxQuant settings were kept the same as default. The MaxQuant search engine Andromeda (Cox et al., 2011) was used with sequence databases *Homo sapiens* (Uniprot 13/01/2013) and *Plasmodium falciparum* 3D7 (PlasmoDB 15/12/2016). A decoy database of reversed sequences was used to filter false positives at a peptide false detection rate of 1%.

The data files generated by MaxQuant were further analyzed using Perseus version 1.5.6.0 (Tyanova et al., 2016b). The list of identified proteins was first filtered to remove potential contaminants, proteins only identified by one site, and proteins identified from the reverse (decoy) peptides. The protein intensity values were then transformed by function $\log_2(x)$. The $\log_2$ intensities were normalized by subtracting the median intensities of each replicate followed by the median intensities of each protein. Proteins identified from fewer than 4 peptides were filtered out at this stage. Student's t-tests were performed to identify proteins with statistically significant changes between conditions using stringency parameters s0 = 0.5 and FDR = 0.01.

Generation of conditional mutant lines

The constructs for the conditional mutation of the catalytic Ser of selected *Pf*SHs (Figure 4A) were based on the pT2A_PfDdi1_cWT construct (kind gift of Dr S. Ridewood, which was in turn based on the pT2A_-FIKK10_cKO construct, kind gift of Dr M. Treeck, The Francis Crick Institute). The plasmid backbone contains a T2A and *gfp* sequence followed by a *P. berghei* dihydrofolate reductase thymidylate synthase for expression of the GOI, and a *hdhfr* cassette to select for transfected parasites with WR99210. The pT2A_-GOI-cMUT plasmids were obtained by combining four DNA fragments via in-fusion PCR using Phusion High-Fidelity DNA Polymerase (New England Biolabs) and the In-Fusion HD Cloning Kit (Clontech) as per manufacturer's instructions. The four DNA fragments containing 15 nucleotides overlapping sequences were: First, the plasmid backbone obtained after digestion of pT2A_PfDdi1_cWT with SalI and BglII (New England Bioscience). Second, the HR upstream of the catalytic Ser that was amplified from *P. falciparum* B11 gDNA purified using a DNeasy Blood and Tissue Kit (Qiagen). The third and fourth fragments were PCR amplified from synthetic DNA (GENEWIZ). The third includes the first loxPint, the RR1 and the triple HA tag, and the fourth the *npt* gene, the second loxPint and the RR2 containing the catalytic Ser to Ala mutation. RR1 was recodonized for *Escherichia coli* and RR2 for *Oncorhynchus mykiss*. Oligonucleotide primers were used at 200 nM (Table S1). PCR and digestion products were purified using QIAquick PCR Purification Kit (Qiagen) as per manufacturer's instructions. Oligonucleotide primers were obtained from Sigma. PCRs were carried out using a thermocycler, and amplicons were analyzed by examining 1% agarose gel electrophoresis stained with SYBR Safe (Thermo Fisher Scientific). DNA concentration was measured using a NanoDrop 2000 spectrophotometer (Thermo Fisher Scientific). pT2A_GOI-cMUT plasmids were amplify in XL10-Gold Ultracompetent cells (Aligent) and purified using QIAprep Midi Kits (Qiagen). Plasmids sequences were checked via capillary sequencing by Source BioScience.

Plasmids were transfected into *P.* falciparum B11 using an Amaxa 4-D electroporator (Lonza) as follows. 10 μg of pT2A_GOI-cMUT plasmid were ethanol precipitated, re-suspended in 10 μL sterile TE buffer (10 mM Tris-HCl pH 8.0, 1 mM EDTA) and mixed with 15–20 μL of Percoll-purified mature schizonts suspended in 100 μL of Amaxa Primary Cell solution P3 (P3 Primary cell 4-D Nucleofector X kit, Lonza). This mixture was electroporated using the FP158 pre-set condition, then immediately transferred to 2 mL of RBCs suspended in complete media (20% hematocrit) and incubated for 30 min at 37°C with shaking to allow egress and invasion to take place. A further 8 mL of complete media was then added, and the cultures were incubated at 37°C under standard conditions overnight. The media was then replaced and supplemented with 2.5 nM WR99210 (WR, Jacobus Pharmaceuticals) to select for transfected parasites. When WR resistant parasites were observed at 1% parasitemia (approximately 2 weeks), Geneticin Selective Antibiotic G418 Sulphate (G418, Gibco) was added at 450 μL/mL to select for chromosomal integration of the plasmid. When 1% parasitemia of WR and G418 resistant parasites was observed (2–4 weeks), samples were taken for cryopreservation, and gDNA extracted to confirm integration by PCR. Clonal lines were obtain using a plaque assay: cultures at 0.75% hematocrit were serially diluted in 96-well flat-bottom plates (Corning Costar) and cultured for 12 days in media containing WR and G418. Plates were then inspected using an inverted light microscope to identify wells containing a single plaque corresponding to a single clonal parasite population. The content of some of these wells (at least 5 per cMUT line) were expanded, and integration verified by PCR. Excision efficiency at the DNA level was measured by diagnostic PCR. Synchronous

cultures of parasites at ring stage were treated overnight with 20 nM rapamycin (RAP, Sigma-Aldrich) or DMSO in media containing WR, and gDNA extracted for diagnosis PCR.

Western blot analysis

Excision efficiency at the protein level was assessed by WB analysis. Synchronous parasite cultures were DMSO or RAP treated as described above, and parasite pellets collected at schizont or merozoite stage. Soluble parasite lysates were separated by SDS-PAGE and transferred to a nitrocellulose membrane using an Appleton Woods wet blotter apparatus. Blots were blocked with 5% BSA (Sigma) in PBST (1% Tween 20 in PBSa) for 2h, followed by 1-h incubation with a 1:1000 dilution of anti-HA antibody (Anti-HA 3F10 monoclonal rat antibody, Roche) in 2% BSA and 3 washes with PBST. The blot was then incubated for 1h with a 1:10,000 dilution of anti-rat-HRP (Goat, polyclonal, anti-rat-HRP, Bio-Rad) in 2% BSA, followed by three washes in PBST. HRP was activated by adding Immobilon Western Chemiluminescent HRP Substrate (Millipore) and visualized using a ChemiDoc Imager (Bio-Rad).

WB analysis of APEH and LYPLA1 was performed as above using 1:500 dilutions of anti-LYPLA1 (Abcam) or anti-APEH (Atlas Antibodies) monoclonal antibodies for 1h. After washing, this was followed by 30 min incubation with anti-rabbit-biotin (Goat, polyclonal, Novex), and finally 30 min with 1/2000 Streptavidin-HRP (Invitrogen).

Parasite replication assay

Synchronous cultures of our cMUT lines were treated with DMSO or RAP at ring stage, diluted to 0.1% parasitemia, and cultured in 96-well round-bottom plates for 3–4 cycles. Samples were collected at schizont stage on the cycle of treatment and at trophozoite stage on the following cycles, fixed for 30 min with 4% paraformaldehyde and 0.02% glutaraldehyde (Sigma) in PBSa at RT, diluted 5-fold in PBSa, and stored at 4°C. To determine the antiparasitic activity of *Hs*SH inhibitors, a synchronous culture of *P. falciparum* 3D7 parasite at 1% parasitemia was treated at ring stage with a dose response of compounds and cultured for 72h in 96-well round-bottom plates. Samples were fixed as described above.

To determine parasitemia by flow cytometry, fixed samples were stained with 1 μg/mL of Hoechst 33342 (Thermo Fisher Scientific) in PBSa for 30 min at 37°C. Five to ten thousand RBCs per samples were analyzed by flow cytometry using a high-throughput Fortessa Analyzer (BD Biosciences) and the FACSDiva Software v8.0.1. Infected RBCs were distinguished from uRBCs as the population that showed positive DNA staining using a 355 nm UV excitation laser and a 450/50 nm emission filter. Flow cytometry data were analyzed using FlowJo LLC 2006–2015. For inhibitor treatment, EC_{50} values were obtained by fitting the parasitemia to a dose response curve in Prism.

Phenotypic characterization of *Hs*SHs inhibitor treatment by flow cytometry

To determine which stage of parasite development was affected by the LYPLA1 and APEH inhibitors ML348 and AA74-1, we used a recently developed flow cytometry assay that allows the dissection of the erythrocytic cycle with hourly resolution. This assay uses highly specific DNA (Hoechst) and RNA (132A, kind gift of Prof Y.T. Chang, National University of Singapore) dyes to monitor intracellular parasite development. Although DNA signal in iRBCs only increases during schizogony because of DNA replication, RNA levels increase throughout the cycle, thus allowing us to monitor the ring to trophozoite transition. In addition, this assay also allows us to monitor egress and invasion given the big difference in DNA and RNA content between mature schizonts and rings.

A synchronous culture of *P. falciparum* 3D7 parasites at 2% parasitemia was treated at ring stage with 3 times the EC_{50} values of ML348 (5 μM) or AA74-1 (0.625 μM). DMSO and CQ (2 μM) were used as negative and positive controls. Samples were collected 24, 41, 48, and 72 h after treatment, fixed and stored as described above, stained for 30 min with 1 μg/mL Hoechst and 2 μM 132A at 37°C, and analyzed by flow cytometry. 132A signal was measured using a 488nm excitation laser and a 610/20 nm emission filter. DNA and RNA levels in iRBCs were calculated as the median DNA and RNA signal of iRBCs divided by that of uRBCs. For those time points in which two distinct populations of iRBCs were observed, generally schizont and ring stage parasites, the DNA and RNA content of these two populations were calculated independently because these populations correspond to iRBCs belong to the first or second cycle after treatment.

QUANTIFICATION AND STATISTICAL ANALYSIS

Growth assay quantification was represented by the mean ± standard deviation as indicated in figure legends and calculations were performed using GraphPad Prism Software. Quantification and statistical analysis of proteomic data was done using MaxQuant version 1.6.2.1 (Tyanova et al., 2016a) and Perseus version 1.5.6.0 (Tyanova et al., 2016b) as described in the LCMS/MS data analysis section of the STAR Methods. The statistical tests used are one-way ANOVA and Student's t-test, as indicated. $p \leq 0.001$, ***; $p \leq 0.01$, **; $p \leq 0.05$, *; $p < 0.05$, non-significant.

iScience, Volume *25*

Supplemental information

Activity-based protein profiling of human and *plasmodium* serine hydrolases and interrogation of potential antimalarial targets

Dara Davison, Steven Howell, Ambrosius P. Snijders, and Edgar Deu

Supplementary Material

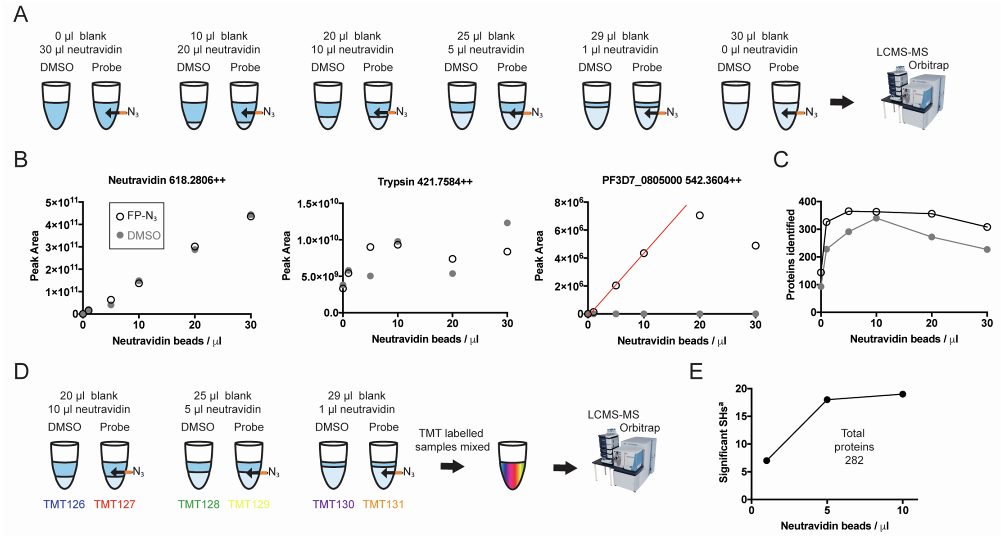

Figure S1. Optimisation of affinity purification for chemical proteomics, related to Figure 2. Contamination from tryptic neutravidin and trypsin peptides is inherent the proteomics methods and can mask identification of lower abundance peptides. We proposed to reduce the ratio of neutravidin to non-neutravidin peptides by optimising the volume of NBs used in order to achieve the highest bead engagement with the lowest bead volume. **(A)** Schizont lysates were labelled in triplicate with 1 μM FP-N_3 or DMSO and treated with CuAAC-chemistry reagents. Labelled proteins were affinity purified using six different NB:blank beads mixtures, followed by on-bread trypsin digestion and LC-MS/MS analysis. **(B)** The raw intensities of specific peptides from neutravidin, trypsin and SHs were assessed using Skyline. In probe-treated samples, neutravidin peptides increased in intensity linearly with NB volume (shown by red line), but the increase in SH peptide intensity slowed above 10 μl NB. Trypsin peptide intensities were not as constant as expected but showed no clear trend. **(C)** Proteins were then quantified using iBAQ and filtered as previously described. In probe-treated samples, the number of total proteins and SHs identified plateaued at 5 μl. Because most SHs were not detected in the DMSO control, the fold enrichment could only be calculated for a small fraction of the SHs identified in the probe-treated samples. **(D)** In order to circumvent the problem of low SH identifications in the control samples and improve our quantification method, we tested TMT isobaric labels to re-quantify the 1, 5 and 10 μl NB samples (TMT-sixplex) as these conditions gave high total protein IDs with low raw intensities of neutravidin peptides (**B-C**). TMT labelled samples are subsequently combined, and the mixture is fractionated by high pH before LCMS-MS. **(E)** Fewer total proteins were identified in the TMT samples, but the number of proteins identified (282) was consistent over all FP-N_3- and DMSO-treated samples, as expected. The highest number of SHs significantly enriched in FP-N_3- vs DMSO-treated samples ([a]student t-test, FDR = 0.05, s_0 = 0.5) was found with 10 μl NB.

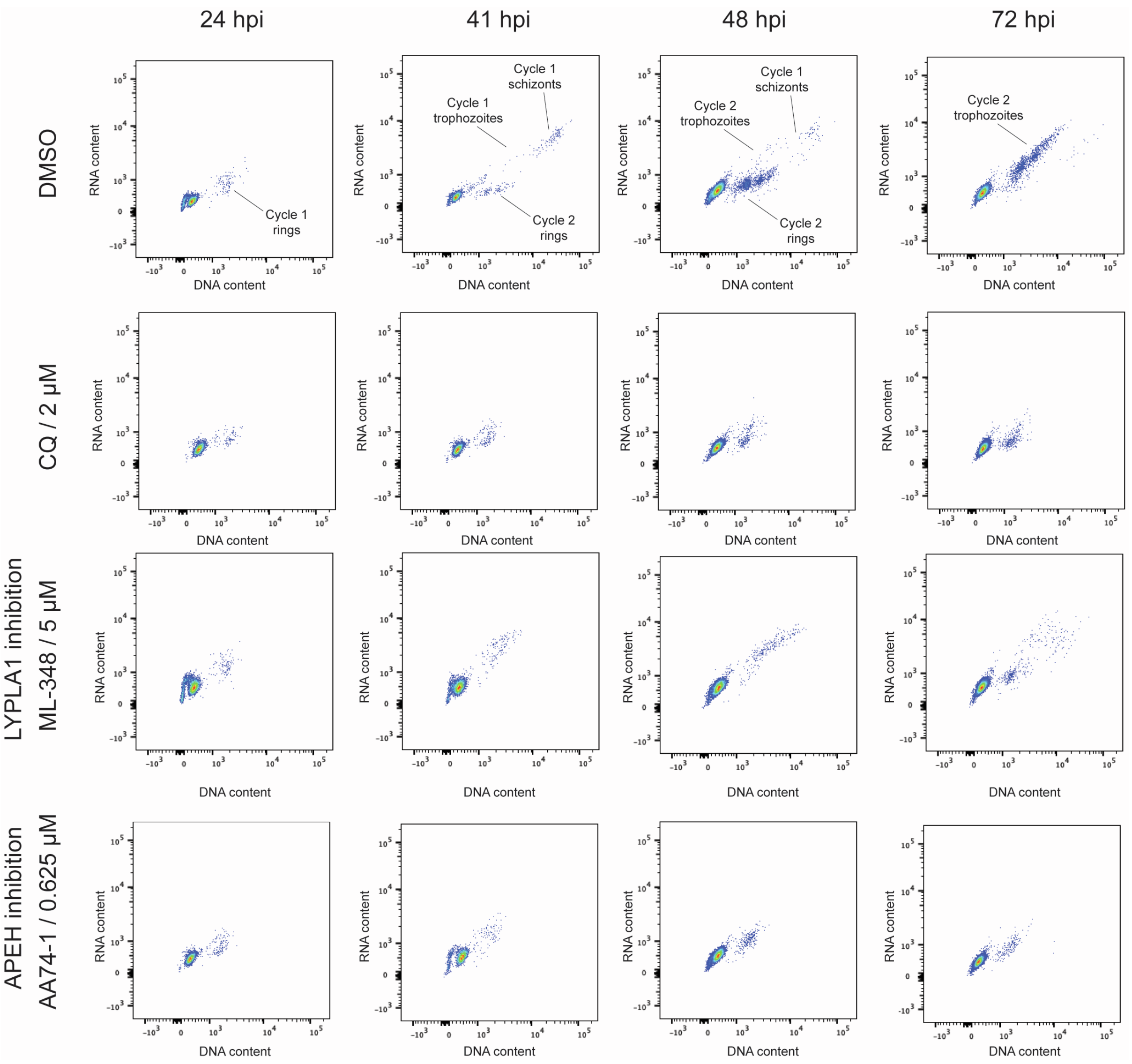

Figure S2. Replication assay to monitor the effect of human SH inhibitors on parasite growth and replication, Related to Figure 5. A growth assay was carried out in the presence of inhibitors ML348 or AA74-1 (3 times the calculated EC_{50}), DMSO and CQ were used as negative and positive controls. Parasites were treated at ring stage (0 hpi). At 24, 41, 48 and 72 hpi, samples were fixed and stained with Hoechst and the RNA dye 132A. This experiment was performed in triplicate. A representative FACS plot for each treatment and time point is shown. Flow cytometry plots show changes in DNA and RNA level overtime for each treatment. The population of RBCs with low DNA and RNA signal correspond to uRBCs. The three different morphological stages of the parasites in iRBCs can be clearly identified as: rings, low DNA and RNA content; trophozoites, low DNA, medium RNA content, and schizonts, high DNA and RNA content. DMSO-treated parasites developed as expected. CQ- and AA74-1-treated samples were arrested at ring stage. ML348-treated parasites appear to still be in trophozoite stage at 48 hpi. While DMSO treated parasite are actively egressing and invading at 48 hpi, no rings were detected in ML438-treated samples until 72 hpi.

Table S1. Activity-based protein profiling across the *Plasmodium falciparum* asexual lifecycle data, Related to Figure 3.
Excel file

**Table S2. Primers for the generation of *P. falciparum* conditional mutant lines, related to Key resource table, Oligonucleotides.**			
**Primer**	**Sequence (5'-3')**	**Source**	**Identifier**
Colony and 3' integration PCR F 119	ACAGCTATGACCATGATTACGC	Sigma	N/A
Colony PCR and synthetic DNA amplification R 256	CGAATAGCCTCTCCACCCAA	Sigma	N/A
Excision PCR R 198	CCTCTCCACTGACAGAAAATTTGTGCC	Sigma	N/A
Synthetic DNA amplification F 257 (also abH114 excision PCR F)	TGGAGAGGCTATTCGGCTATGAC	Sigma	N/A
abH112 3' integration PCR R 296	AGCATTTTCAACTCCCCAGAATCGAAG	Sigma	N/A
abH112 Amplification and mutation R 270	CTTCCTCTTCCTTCTCCGTCGACCTCGTTC	Sigma	N/A
abH112 Endogenous PCR F 315	CATTCTTGGTTGTATGACCATATGGTAG	Sigma	N/A
abH112 Endogenous PCR R 317	ATTCTATATAATTCATGTAACAGTAACATC	Sigma	N/A
abH112 Excision PCR F 295	GAATACTACAGATATTGGTTATATGACAC	Sigma	N/A
abH112 HR amplification F 284	AGACTTAGGAGGAGATCTGTAGCGGGTTTA GGGGACGCATGGTGAA	Sigma	N/A
abH112 HR amplification R 269	CTTAATCCATAACCACTATAATCATATGAA	Sigma	N/A
abH112 Mutation PCR amplification 2/2 SER a R 278	CCAAGAGAATGTCCATAAACAATAATATTC	Sigma	N/A
abH112 Mutation PCR amplification 2/2 SER b F 277	TGGACATTCTCTTGGATCTGCAACCAGTTGT TA	Sigma	N/A
abH112 Synthetic DNA amplification ½ F 271	GTGGTTATGGATTAAGTAATAAAGATCCTAG TG	Sigma	N/A
abH114 3' integration PCR R 299	GGAATTCTCTAAAATGATTTGGCTAGCTGAA GG	Sigma	N/A
abH114 Amplification and mutation R 276	AATAAACTTCCTCTTCCTTCTCCGTCGACTT TG	Sigma	N/A
abH114 Endogenous PCR F 319	CAGTTATATACCTTTATCAGGTGTGT	Sigma	N/A
abH114 Endogenous PCR R 321	CATACATATAAATCTTCTTTATTATGC	Sigma	N/A
abH114 HR amplification F 283	AGACTTAGGAGGAGATCTTCCCCTTATAAAT CTTTCAGAAAGAACGTC	Sigma	N/A
abH114 HR amplification R 274	ATCGCATATTTTCGGTATGCTTAGAATC	Sigma	N/A
abH114 Mutation PCR amplification 2/2 SER a R 281	CCTCCTAAAGAGAATCCAATTAAGCAAC	Sigma	N/A
abH114 Mutation PCR amplification 2/2 SER b F 280	GATTCTCTTTAGGAGGAAAAGCCTCTAT	Sigma	N/A
abH114 Synthetic DNA amplification ½ F 275	CCGAAAATATGCGATTTGATGAAATTG	Sigma	N/A
Psta1 5' integration PCR R 291	GTCCATATTCTCCAATGTATATATCTCC	Sigma	N/A
Psta1 Amplification and mutation R 235	TTCCTTCTCCGTCGACTTCA	Sigma	N/A
Psta1 Endogenous PCR R 309	CATTTAATTATATTCTAAAGACTTTG	Sigma	N/A
Psta1 Endogenous/5' excision PCR F 307	CTTACTATAATAATAAAGTGAACACACA	Sigma	N/A
Psta1 Excision PCR F 289	AGTACATGGGTTAGCTTCTCATATAAGAT	Sigma	N/A
Psta1 HR amplification F 218	ACTTAGGAGGAGATCTAATAATAAAAATGAT ATAACCATTGATGAGAA	Sigma	N/A
Psta1 HR amplification R 225	CTATAATATCGTTATCTCTTATATACATTTGT TT	Sigma	N/A
Psta1 Mutation PCR amplification 2/2 SER a R 240	ATACTATAACCAACAAGGTATATTGGTA	Sigma	N/A
Psta1 Mutation PCR amplification 2/2 SER b F 239	TTGGTTATAGTATGGGAGCTAATATAATACT TAGGGC	Sigma	N/A
Psta1 Synthetic DNA amplification ½ F 223	ATAACGATATTATAGGTAAATAAAAAAAAATA ATATACAATAACTTCGTATAGCATACAT	Sigma	N/A
Psta2 5' integration PCR R 294	CTTAGTTATTGTACCGTCGTACCTCACTTT	Sigma	N/A
Psta2 Amplification and mutation R 236	TTCCTTCTCCGTCGACTTTT	Sigma	N/A
Psta2 Endogenous PCR R 313	AAACGATTCAGTGTAGACAAA	Sigma	N/A
Psta2 Endogenous/5' excision PCR F 311	TTACACTGTTGTATAAGTTTGAA	Sigma	N/A
Psta2 Excision PCR F 292	TACATGGGTTAGCTTCTCATTTAAGG	Sigma	N/A
Psta2 HR amplification F 220	ACTTAGGAGGAGATCTACAGAAAGTAATATT ATTAATGATGAGAA	Sigma	N/A
Psta2 HR amplification R 229	CTGCTCCTTATCTAATGTGTCACTTCTCGTT TC	Sigma	N/A
Psta2 Mutation PCR amplification 2/2 SER a R 242	ACCCATATCAAAACCAGCCAAGTAAATTG	Sigma	N/A
Psta2 Mutation PCR amplification 2/2 SER b F 241	GGTTTTAGTATGGGTGCCAATATTATGTTAC G	Sigma	N/A
Psta2 Synthetic DNA amplification ½ F 232	TTAGATAAGGAGCAGGTAAATAAAAAAAATA ATATACAATAACTTCGTATAGCATACAT	Sigma	N/A
